# FBXO28 promotes cell proliferation, migration and invasion via upregulation of the TGF-beta1/SMAD2/3 signaling pathway in ovarian cancer

**DOI:** 10.1186/s12885-024-11893-8

**Published:** 2024-01-24

**Authors:** Gendi Song, Zhengwei Sun, Man Chu, Zihan Zhang, Jiajia Chen, Zhiwei Wang, Xueqiong Zhu

**Affiliations:** Zhejiang Provincial Clinical Research Center for Obstetrics and Gynecology, Department of Obstetrics and Gynecology, The Second Affiliated Hospital of Wenzhou Medical University, Wenzhou Medical University, Wenzhou, 325027 China

**Keywords:** FBXO28, TGF-b1, Ovarian cancer, Smad2/3, Migration

## Abstract

**Background:**

Ovarian cancer is one of the most common gynecological malignancies due to the lack of early symptoms, early diagnosis and limited screening. Therefore, it is necessary to understand the molecular mechanism underlying the occurrence and progression of ovarian cancer and to identify a basic biomarker for the early diagnosis and clinical treatment of ovarian cancer.

**Methods:**

The association between FBXO28 and ovarian cancer prognosis was analyzed using Kaplan‒Meier survival analysis. The difference in FBXO28 mRNA expression between normal ovarian tissues and ovarian tumor tissues was obtained from The Cancer Genome Atlas (TCGA), and Genotype-Tissue Expression (GTEx) cohorts. The expression levels of the FBXO28 protein in ovarian cancer tissues and normal ovarian tissues were measured via immunohistochemical staining. Western blotting was used to determine the level of FBXO28 expression in ovarian cancer cells. The CCK-8, the colony formation, Transwell migration and invasion assays were performed to evaluate cell proliferation and motility.

**Results:**

We found that a higher expression level of FBXO28 was associated with poor prognosis in ovarian cancer patients. Analysis of the TCGA and GTEx cohorts showed that the FBXO28 mRNA level was lower in normal ovarian tissue samples than in ovarian cancer tissue samples. Compared with that in normal ovarian tissues or cell lines, the expression of FBXO28 was greater in ovarian tumor tissues or tumor cells. The upregulation of FBXO28 promoted the viability, proliferation, migration and invasion of ovarian cancer cells. Finally, we demonstrated that FBXO28 activated the TGF-beta1/Smad2/3 signaling pathway in ovarian cancer.

**Conclusions:**

In conclusion, FBXO28 enhanced oncogenic function via upregulation of the TGF-beta1/Smad2/3 signaling pathway in ovarian cancer.

**Supplementary Information:**

The online version contains supplementary material available at 10.1186/s12885-024-11893-8.

## Background

Ovarian cancer (OC) is one of the most common tumors in females worldwide and has a high incidence and poor prognosis [1, 2]. The number of deaths from ovarian cancer will increase to 254,000 in 2035 [3]. Approximately 80% of ovarian cancer patients are diagnosed at advanced stages, resulting in an overall 5-year survival rate of 30–50% [4]. This poor survival rate is largely attributed to the absence of early symptoms, limited detection of biomarkers, and a shortage of diagnostic approaches [5, 6]. Hence, it is urgent to enhance the understanding of the molecular mechanisms of ovarian cancer and develop novel clinical treatment strategies to improve the outcome of patients with this disease [7–9].

The F-box proteins consist of three subfamilies: F-box proteins with leucine-rich amino acid repeats (FBXL), F-box proteins with WD40 amino acid repeats (FBXW), and F-box proteins with uncharacterized domains only (FBXO) [10]. Generally, F-box proteins can directly bind to substrates and carry out proper posttranslational modifications via ubiquitination and subsequent substrate degradation [11]. F-box proteins play a critical role in tumor development and progression via the regulation of proliferation, apoptosis, epithelial–mesenchymal transition (EMT), cancer stem cells and drug resistance [12–15]. FBXO28 has been reported to participate in oncogenesis in a variety of human cancers. For example, one study showed that 6-phosphofructo-2-kinase/fructose-2, 6-biphosphatase 4 (PFKFB4) binds to FBXO28 to regulate the ubiquitination and proteasomal degradation of HIF-1α, promoting HIF-1α signaling in glioblastoma [16]. Another study revealed that FBXO28 silencing enhances pancreatic β-cell apoptosis and induces diabetes mellitus [17]. In addition, FBXO28 delivers CDK activity to MYC, affecting the cell cycle of breast cancer cells, which is associated with the poor prognosis [18]. However, the role of FBXO28 in ovarian carcinogenesis is largely elusive.

The TGF-b superfamily includes a wide range of proteins, such as TGF-b1, TGF-b2, TGF- b3, bone morphogenetic proteins (BMPs), and some growth and differentiation factors (GDFs). Each member of the superfamily has unique functions and contributes to numerous biological processes [19]. TGF-b family members activate the Smad signaling pathway through the TGF-b receptor (TGF-b RI) and TGF-b RII complex, suggesting that TGF-b RI and TGF-b RII are the main effector proteins in the TGF-b signaling pathway [20, 21]. For example, TGF-b RI activates Smad2 and Smad3, leading to phosphorylation of Smad2 and Smad3 [22]. As a common molecular mechanism, the TGF-b1 signaling pathway is involved in the development of ovarian cancer [23]. For example, TGF-b governs cell invasion via upregulation of CAF-derived versican in the tumor microenvironment in ovarian cancer [24]. Platelets trigger EMT and enhance invasion via the TGF-b1 signaling pathway in ovarian cancer [25]. NR2F1 influences platinum sensitivity and the immune response through the regulation of TGF-b1-mediated EMT in ovarian cancer [26]. However, whether FBXO28 can regulate TGF-b1 during ovarian cancer progression is unclear. Therefore, in the present study, we aim to explore the functions of FBXO28 on cell proliferation, migration and invasion in ovarian cancer. Moreover, we plan to determine whether FBXO28 can promote ovarian oncogenesis via regulation of TGF-b1 and SMAD2/3 signaling pathways in ovarian cancer. Our study demonstrated that FBXO28 facilitates malignant behavior of ovarian cancer cells via activation of TGF-b1 /SMAD2/3 pathway.

## Materials and methods

### Database analysis

Ovarian cancer data were obtained from The Cancer Genome Atlas (TCGA) database (https://portal.gdc.cancer.gov/), and normal ovarian data were obtained from The Cancer Genome Atlas (GTEx) (https://www.gtexportal.org/home/). The TCGA database has no normal ovarian data. The KEGG database (https://www.genome.jp/kegg) was used to determine signaling pathways that were associated with enrichment of FBXO28.

### Cell culture

The normal ovarian cell line IOSE80 (Shanghai Cell Bank, Chinese Academy of Sciences) and ovarian cancer cells, including SKOV3, OVCAR3 and A2780 cells (Shanghai Cell Bank, Chinese Academy of Sciences), were used in this study. IOSE80, SKOV3 and OVCAR3 cells were cultured in Roswell Park Memorial Institute (RPMI) 1640 medium (Gibco, USA) supplemented with 10% fetal bovine serum (FBS; Gibco, USA) and 1% penicillin‒streptomycin (Meiluncell, Dalian). A2780 cells were cultured in DMEM medium (Gibco, USA) supplemented with 10% FBS and 1% penicillin‒streptomycin. The cells were cultured at 37 ℃ in a 5% carbon dioxide humidification incubator.

### Tissue samples

Ten human ovarian cancer tissue samples and 10 human normal ovarian tissue samples were collected from different patients diagnosed and pathologically confirmed at the Second Affiliated Hospital of Wenzhou Medical University (Supplementary Table 1). This study was approved by the Ethics Committee of the Second Affiliated Hospital of Wenzhou Medical University (Ethics Committee Approval Number: 2023-K-05-01).

### Immunohistochemical staining (IHC)

The paraffin sections containing tissues were placed in a drying oven at 60 ℃ for 2 h to finish baking the slices, which were subsequently dewaxed with xylene and gradient ethanol. The tissues were soaked in sodium citrate buffer solution with a heated boil, after which antigen retrieval was completed. The endogenous enzyme activity was inactivated by hydrooxidase, 5% bovine serum albumin (BSA) was used to block the tissue sections, which were incubated overnight at 4 ℃ with primary antibodies, including rabbit anti-FBXO28 antibody (1:100; Abcam), rabbit anti-N-cadherin antibody (1:200; Proteintech), rabbit anti-E-cadherin antibody (1:100; Proteintech) and mouse anti-Ki67 antibody (Zsbio, China). After the sections were incubated at room temperature with the corresponding secondary antibodies, DAB and hematoxylin were used to stain the sections. The staining intensity was scored according to the following criteria: 0 (blue, feminine), 1 (light yellow, weakly positive), 2 (brownish, yellow, moderately positive), or 3 (dark brown, strong positive). The staining area was scored as 1 (≤ 25% of positive cells), 2 (26–50% of positive cells), 3 (51–75% of positive cells), or 4 (≥ 75% of positive cells). The IHC staining was calculated by multiplying the staining intensity score and positive staining percentage score [27].

### Western blotting analysis

Total protein was extracted by using RIPA lysis buffer containing 1% phenylmethanesulfonyl fluoride (PMSF) (Beyotime, Shanghai, China) after being lysed on ice, ultrasonicated and centrifuged. Based on the protein concentration determined by the BCA protein detection kit (Beyotime, China), an equally concentrated protein mixture was prepared with loading buffer solution (Biosharp, Hefei, China). The target proteins were separated via 8% or 10% SDS‒PAGE gel. Then proteins were subsequently transferred to a PVDF membrane at 300 mA for 90 min to complete the transmembrane. After 5% skim milk powder was added for 1 h, the membrane was incubated with primary antibodies at 4 ℃ overnight, which include rabbit anti-FBXO28 antibody (1:1000; abcam), rabbit anti-TGF-b1antibody (1:1000; abways), rabbit anti-Smad2/3 antibody (1:1000; CST), rabbit anti-p-Smad2/3 antibody (1:1000; CST), rabbit anti-N-cadherin antibody (1:1000; Proteintech), rabbit anti-E-cadherin antibody (1:1000; Proteintech), mouse anti-GAPDH antibody (1:5000; Proteintech) and rabbit anti-β-Tubulin (1:2000; Proteintech). After the corresponding secondary antibody was added and incubated at room temperature for 1 h, the proteins were visualized via an enhanced chemiluminescence kit (Meilunbio, China).

### Transfection

The FBXO28 plasmid (GeneCopoeia, China), TGF-b1 plasmid (MiaoLing Biology, China), shFBXO28 plasmid (Genechem, China) and control plasmid (GeneCoopoeia, China) were used for transfection. For lentivirus transfection, HEK293T cells were used as tool cells to obtain viral supernatant via plasmids. The transfection process was completed by treating the targeted cells with the virus supernatant. Purinomycin was used to screen out the resistant cells and to obtain stable cell lines. For liposome transfection, the plasmid was added to the targeted cells to complete the transfection, and the needed resistant cells were generated with neomycin to obtain the stable cell lines.

### Colony formation assay

A total of 500 A2780 cells were inoculated in 6-well plates with complete medium and cultured at 37 ℃ in a 5% carbon dioxide incubator. The cells were grown in clumps for several days. When visible colonies had formed, the cells were stained with fixed crystal violet and 4% paraformaldehyde, photographed and counted.

### CCK-8 assay

SKOV3 and A2780 cells were cultured in complete medium to a density of 2 × 10^3^ cells/ml, and after which 100 µl of solution was subsequently absorbed to the 96-well plate. On days 0, 1, 2, 3, and 4, CCK-8 solution and serum-free and penicillin‒streptomycin media were added to the wells at a volume of 1:10, and 100 µl was added to each well to replace the original medium. The cells were incubated in a 37 ℃ incubator for 2 h, after which the absorbance at a wavelength of450 nm was measured by an enzyme-labeling instrument to determine cell viability.

### Transwell assays for migration and invasion

For the migration assay, 2 × 10^4^ SKOV3 cells and 8 × 10^4^ A2780 cells were inoculated in the upper Transwell chambers (Constar, Corning, USA) containing serum-free medium (Costa, Corning, USA), and the lower chambers were supplemented with 800 µl of 10% serum medium. For the invasion assay, the number of 5 × 10^4^ SKOV3 and 1.5 × 10^5^ A2780 cells were inoculated in the upper Transwell chambers with pre-added Matrigel (BD, USA) supplemented with serum-free medium, and the lower chambers were supplemented with 800 µl of 10% serum medium. After different time, the cells that had passed through the polycarbonate membrane were fixed with 4% paraformaldehyde, stained with crystal violet, photographed, and counted in three fields with a microscope (Leica Germany).

### Animal experiment

All animal study methods were approved by the Animal Care and Use Committee of Wenzhou Medical University and carried out in accordance with relevant guidelines and regulation. Animal xenograft experiments were carried out in accordance with ARRIVE guidelines. The mouse study was conducted on BALB/c nude mice (Beijing Weitong Lihua Scientific Co., Ltd., Beijing, China) aged 4–6 weeks. FBXO28-knockdown or control tumor models were established by subcutaneous injection of 3 × 10^6^ SKOV3 cells with FBXO28 knockdown or control SKOV3 cells suspended in 100 µL of PBS into each nude mouse. The tumor volume was measured every 4 days after tumor formation, and the tumor volume was calculated according to the formula length × width^2^/2. Tumor xenografts were collected from nude mice after 34 days to measure tumor weight.

### Statistical analysis

The experimental data were analyzed using GraphPad Prism 8.0 and are expressed as the mean ± standard deviation. Comparisons between two groups were performed by using a two-sided Student’s t test. Comparisons between multiple groups were performed by one-way ANOVA. *p* < 0.05 was considered to indicate statistical significance.

## Results

### FBXO28 is highly expressed in ovarian cancer tissues and cells

**Fig. 1:**
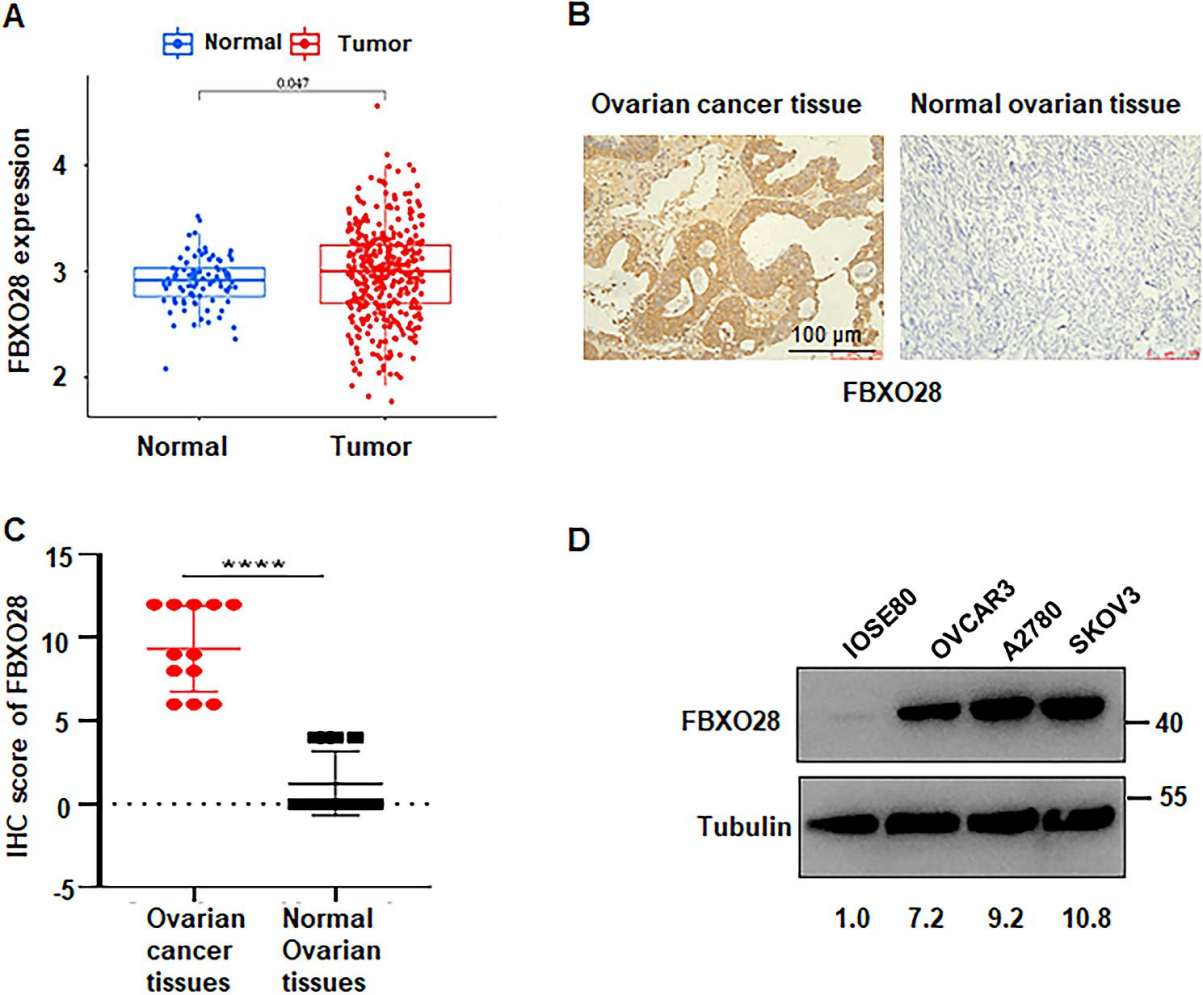
FBXO28 expression is correlated with ovarian cancer prognosis. A. TCGA combined with GTEx analysis showed higher expression levels of FBXO28 in ovarian cancer tissues than in normal ovarian tissues. B. Representative images of immunohistochemical staining for FBXO28 in ovarian cancer and normal ovarian tissue. Scale bar: 100 μm. C. IHC staining results for FBXO28 in ovarian cancer tissues and normal ovarian samples. D. Expression level of FBXO28 in normal ovarian cells and ovarian cancer cells determined by Western blotting

To clarify the role of FBXO28 in ovarian cancer, first, the expression of FBXO28 in normal ovarian tissues and ovarian cancer tissues was analyzed via the GTEx database combined with TCGA data. The results showed that the expression level of FBXO28 in ovarian cancer tissue was greater than that in normal ovarian tissue (*P* < 0.05) (Fig. 1A). Subsequently, we analyzed the expression of the FBXO28 protein in ovarian cancer tissues and human normal ovarian tissues by immunohistochemistry. The results showed that the expression of the FBXO28 protein in ovarian tumor tissues was greater than that in normal ovarian tissues (Fig. 1B-C). In addition, the expression of the FBXO28 protein in normal ovarian IOSE80 cells and ovarian cancer cell lines, including the A2780, SKOV3 and OVCAR3 cell lines, was detected by Western blotting. We found that FBXO28 was expressed at a greater level in ovarian cancer cells than in normal ovarian IOSE80 cells (Fig. 1D). These results suggested that FBXO28 is highly expressed in ovarian cancer cells and tissues.

### FBXO28 overexpression promotes the viability of ovarian cancer cells

**Fig. 2:**
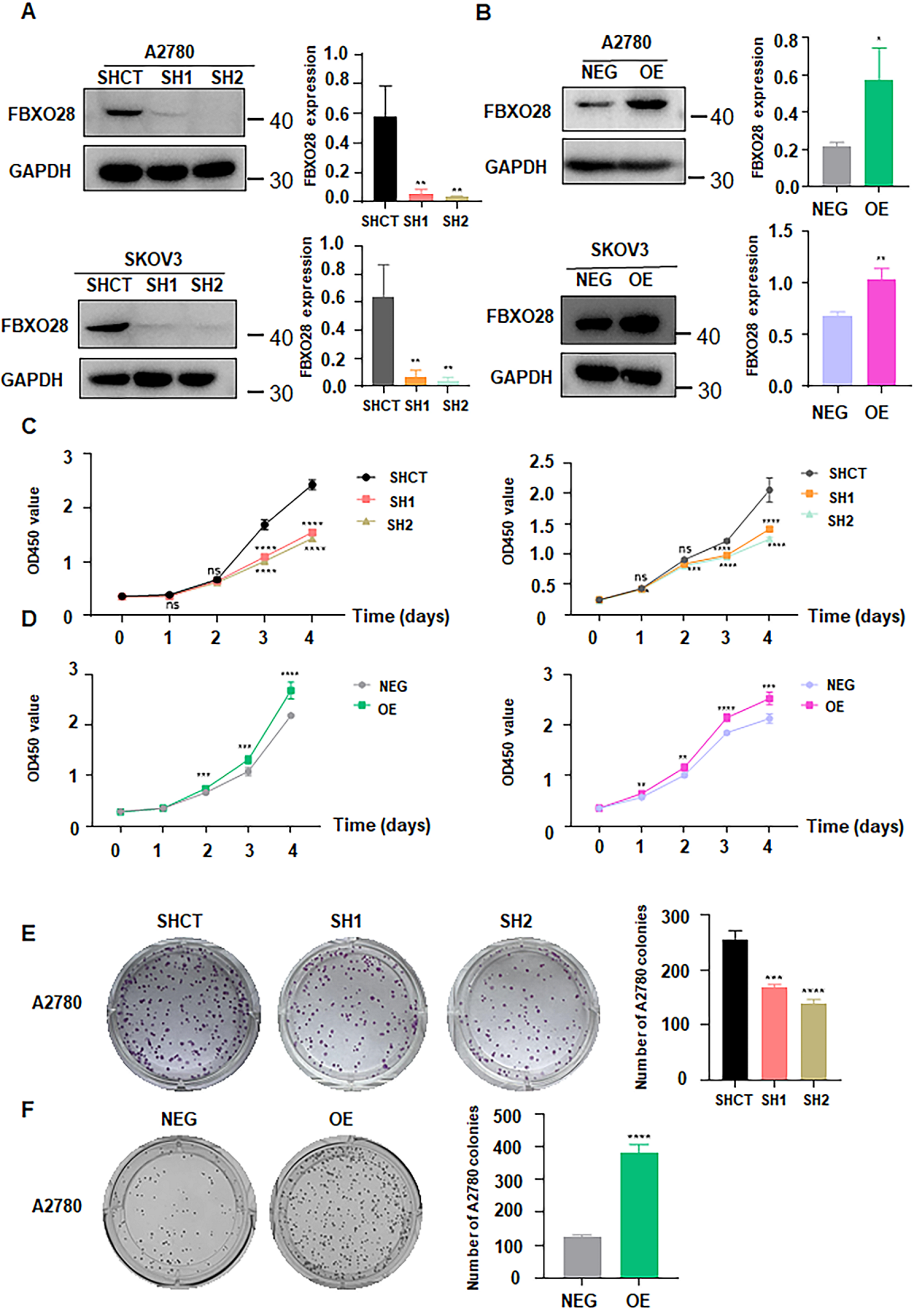
FBXO28 overexpression promotes the viability of ovarian cancer cells. A. Left panel: The efficacy of FBXO28 knockdown was determined using Western blotting. Right panel: Corresponding quantitative analysis results. B. Left panel: The efficacy of FBXO28 overexpression was determined using Western blotting. Right panel: Corresponding quantitative analysis results. C. A CCK-8 assay showed that FBXO28 knockdown suppressed the viability of ovarian cancer cells. D. A CCK-8 assay showed that FBXO28 overexpression promoted the viability of ovarian cancer cells. E. Left panel: FBXO28 knockdown decreased the proliferation capacity of A2780 cells, as determined by a colony formation assay. Right panel: Corresponding quantitative analysis results. F. Left panel: FBXO28 overexpression increased the proliferation capacity of A2780 cells, as determined by a colony formation assay. Right panel: Corresponding quantitative analysis results. *P < 0.05, **P < 0.01, ***P < 0.001, ****P < 0.0001

To further explore the role of FBXO28, we upregulated and downregulated the expression of FBXO28 in A2780 and SKOV3 cells via transfection with the FBXO28-overexpressing plasmid (OE) and the shFBXO28 plasmid, respectively. Western blotting was used to verify the transfection efficacy. We found that both shRNA1 and shRNA2 transfection reduced FBXO28 protein expression in SKOV3 and A2780 cells (Fig. 2A). OE transfection elevated the expression level of the FBXO28 protein in both the SKOV3 and A2780 cell lines (Fig. 2B).

To study the effect of FBXO28 on cell viability and proliferation, we conducted CCK-8 and cell colony formation assays in SKOV3 and A2780 cells after FBXO28 was knocked down or overexpressed. We found that downregulation of FBXO28 inhibited the viability of SKOV3 and A2780 cells (Fig. 2C), while overexpression of FBXO28 promoted ovarian cancer cell viability (Fig. 2D, supplementary Table 2). Colony formation data revealed that overexpression of FBXO28 enhanced the proliferation of A2780 cells, whereas depletion of FBXO28 decreased cell proliferation (Fig. 2E-F, supplementary Fig. 1). These results suggested that FBXO28 is involved in the viability and proliferation of ovarian cancer cells.

### FBXO28 overexpression promotes the migration and invasion of ovarian cancer cells

**Fig. 3:**
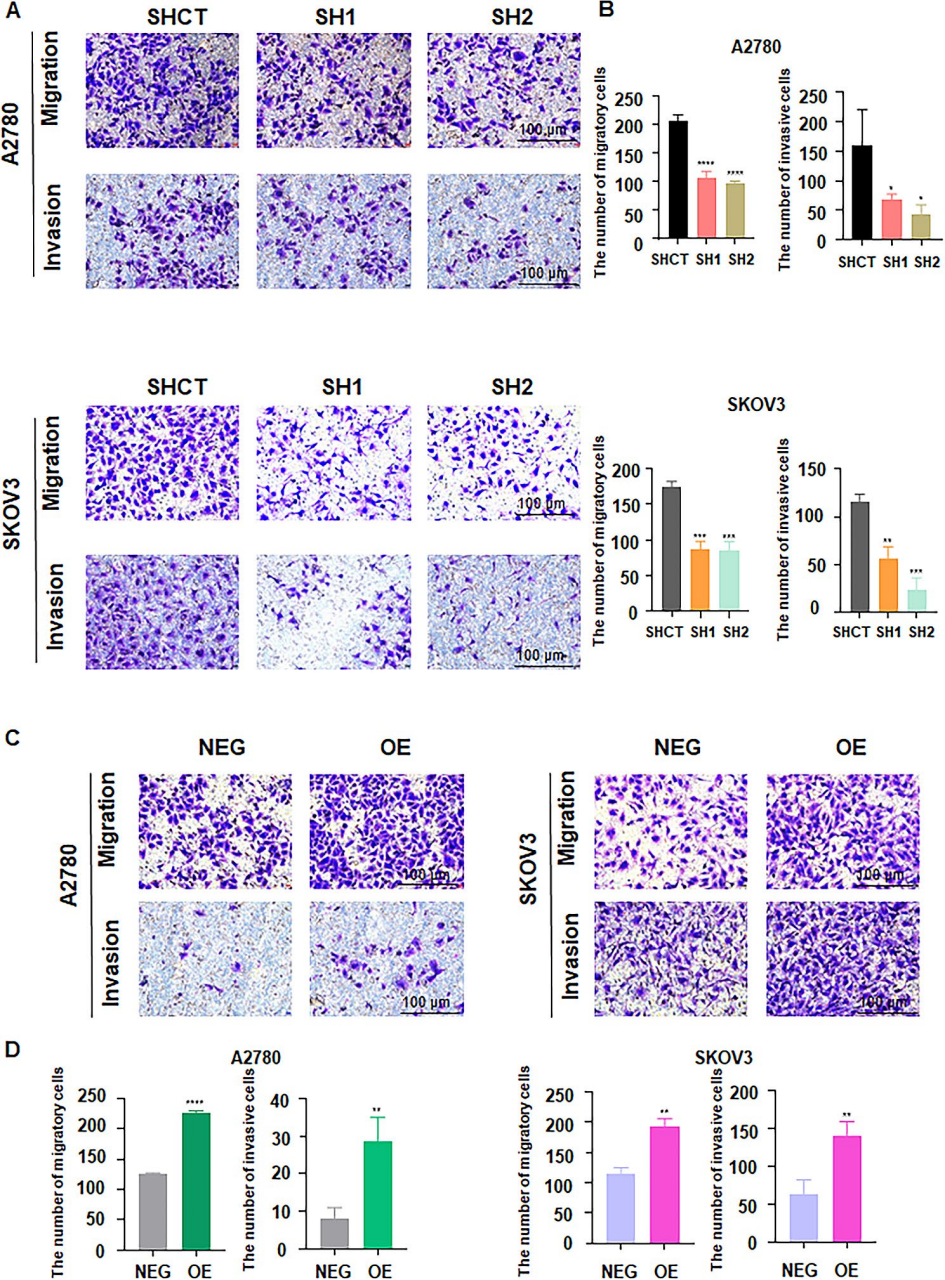
FBXO28 promotes the migration and invasion of ovarian cancer cells. A. Transwell migration and invasion assays showed that knockdown of FBXO28 after 36 h inhibited the migration and invasion of ovarian cancer cells. B. The quantitative analysis results obtained from Figure A. C. Transwell migration and invasion assays showed that overexpression of FBXO28 after 24 h promoted the migration and invasion of ovarian cancer cells. D. The quantitative analysis results obtained from Figure C. *P < 0.05, **P < 0.01, ***P < 0.001, ****P < 0.0001

Next, we investigated whether a change in FBXO28 expression affects the migration and invasion ability of ovarian cancer cells. Transwell migration and invasion assays were performed in SKOV3 and A2780 cells after FBXO28 was knocked down or overexpressed. We observed that downregulation of FBXO28 led to reduced migration and invasion of A2780 and SKOV3 cells (Fig. 3A-B). Consistent with these findings, the migration and invasion abilities of A2780 and SKOV3 cells overexpressing FBXO28 were enhanced (Fig. 3C-D). Thus, our data suggested that FBXO28 participates in promoting the migration and invasion of ovarian cancer cells.

### FBXO28 regulates the TGF-b1/Smad2/3 pathway in ovarian cancer cells

**Fig. 4:**
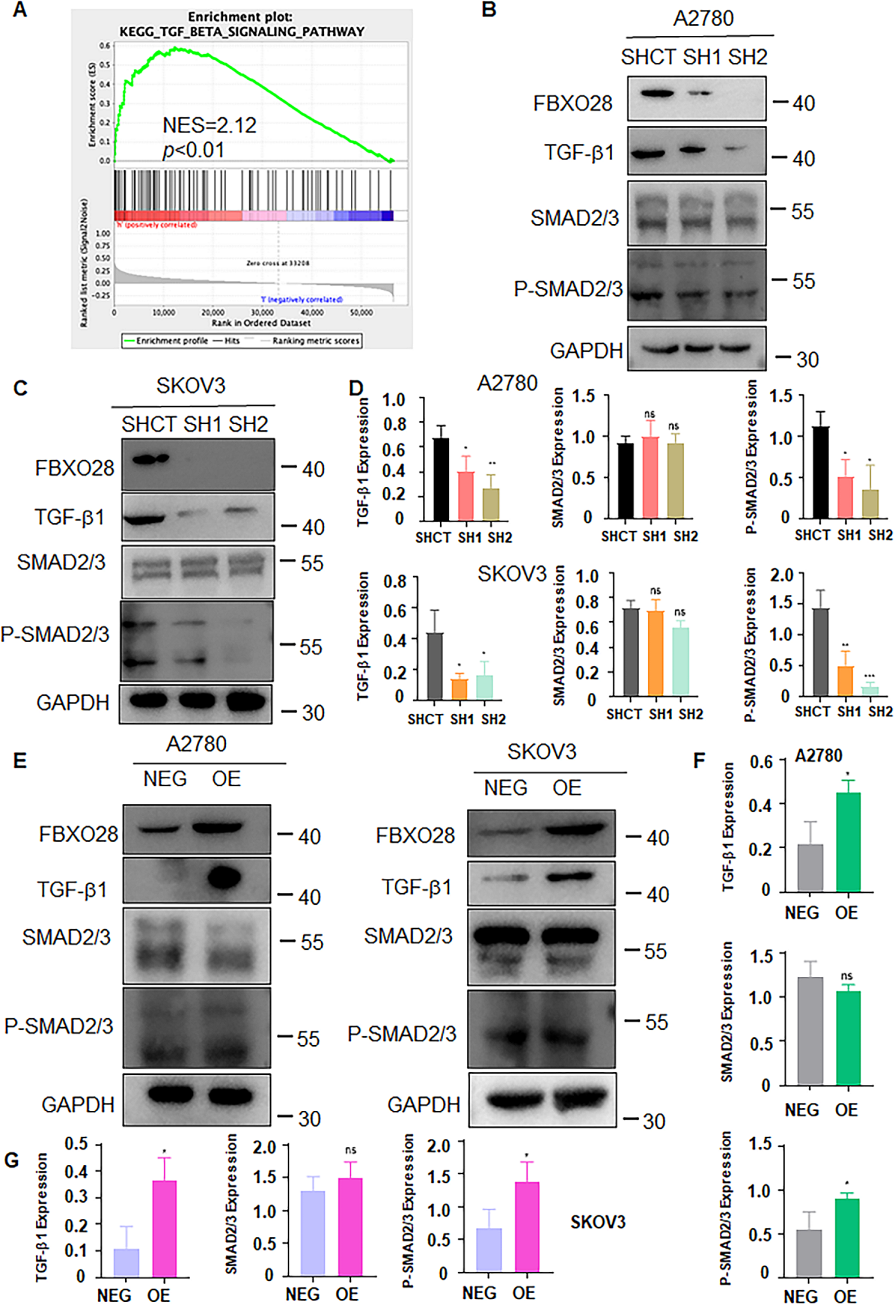
FBXO28 regulates TGF-b1/Smad2/3 pathway in ovarian cancer cells. A. KEGG enrichment analysis showed that FBXO28 may act on ovarian cancer via the TGF-b1 signaling pathway. B. Western blot analysis of TGF-b1 pathway expression after FBXO28 was knocked down in A2780 cells. C. Western blot analysis of TGF-b1 pathway expression after FBXO28 was knocked down in SKOV3 cells. D. The quantitative analysis results obtained in Figure B and C. E. Western blot analysis of TGF-b1 pathway expression after overexpression of the FBXO28 gene in ovarian cancer cells. F-G. The quantitative analysis results for Figure E. *P < 0.05, **P < 0.01, ***P < 0.001

**Fig. 5:**
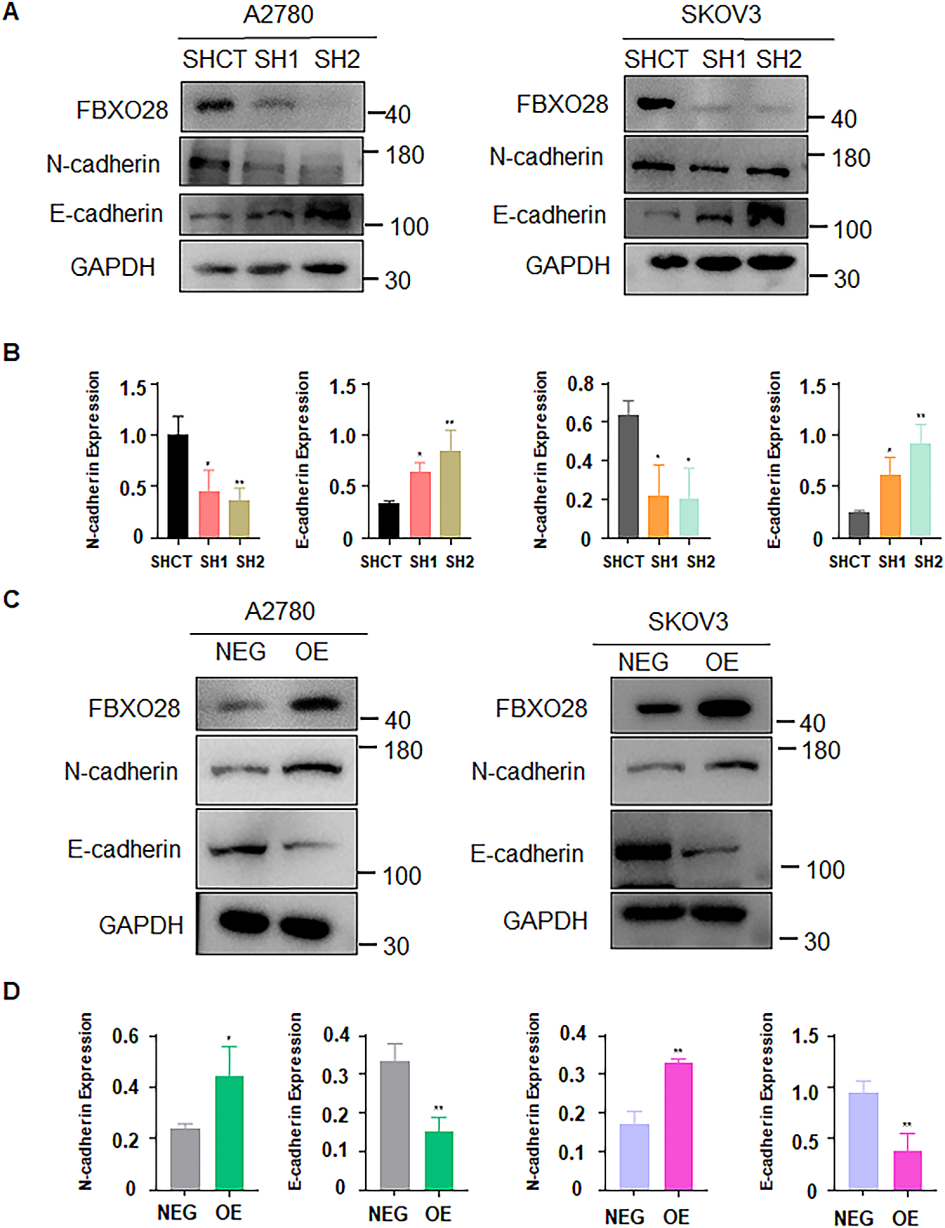
FBXO28 regulates the expression of EMT markers. A. Western blot analysis of EMT marker expression after FBXO28 was knocked down in ovarian cancer cells. B. The quantitative analysis results obtained in Figure A. C. Western blotting analysis of EMT marker expression after FBXO28 overexpression in ovarian cancer cells. D. The quantitative analysis results obtained in Figure C. *P < 0.05, ** P < 0.01

According to the KEGG database, FBXO28 can participate in tumorigenesis by regulating the TGF-b1 signaling pathway (Fig. 4A). Next, we examined whether the TGF-b1 signaling pathway was altered in ovarian cancer cells after FBXO28 modulation. In A2780 and SKOV3 cells, knockdown of FBXO28 reduced the protein expression levels of TGF-b1 and p-Smad2/3 (Fig. 4B-D), while overexpression of FBXO28 increased the protein expression levels of TGF-b1 and p-Smad2/3 (Fig. 4E-G). Numerous studies have shown that TGF-b1 signaling governs the migration and invasion capacity of many human cancers by affecting the expression of EMT indicators, such as breast cancer [28], lung cancer [29], ovarian cancer [26], and prostate cancer [30], by affecting the expression of EMT indicators. Hence, we measured the expression of EMT markers, including E-cadherin and N-cadherin, in cells with altered FBXO28 expression. Western blotting analysis revealed that FBXO28 knockdown increased the protein expression of E-cadherin in A2780 and SKOV3 cells but decreased the protein expression of N-cadherin (Fig. 5A-B). Similarly, after the overexpression of FBXO28, N-cadherin protein expression was increased, while E-cadherin protein expression was decreased in A2780 and SKOV3 cells (Fig. 5C-D). Therefore, FBXO28 may regulate the expression levels of EMT factors through the TGF-b1 signaling pathway and affect the progression of ovarian cancer.

### TGF-b1 overexpression rescues FBXO28 knockdown-mediated antitumor activities

**Fig. 6:**
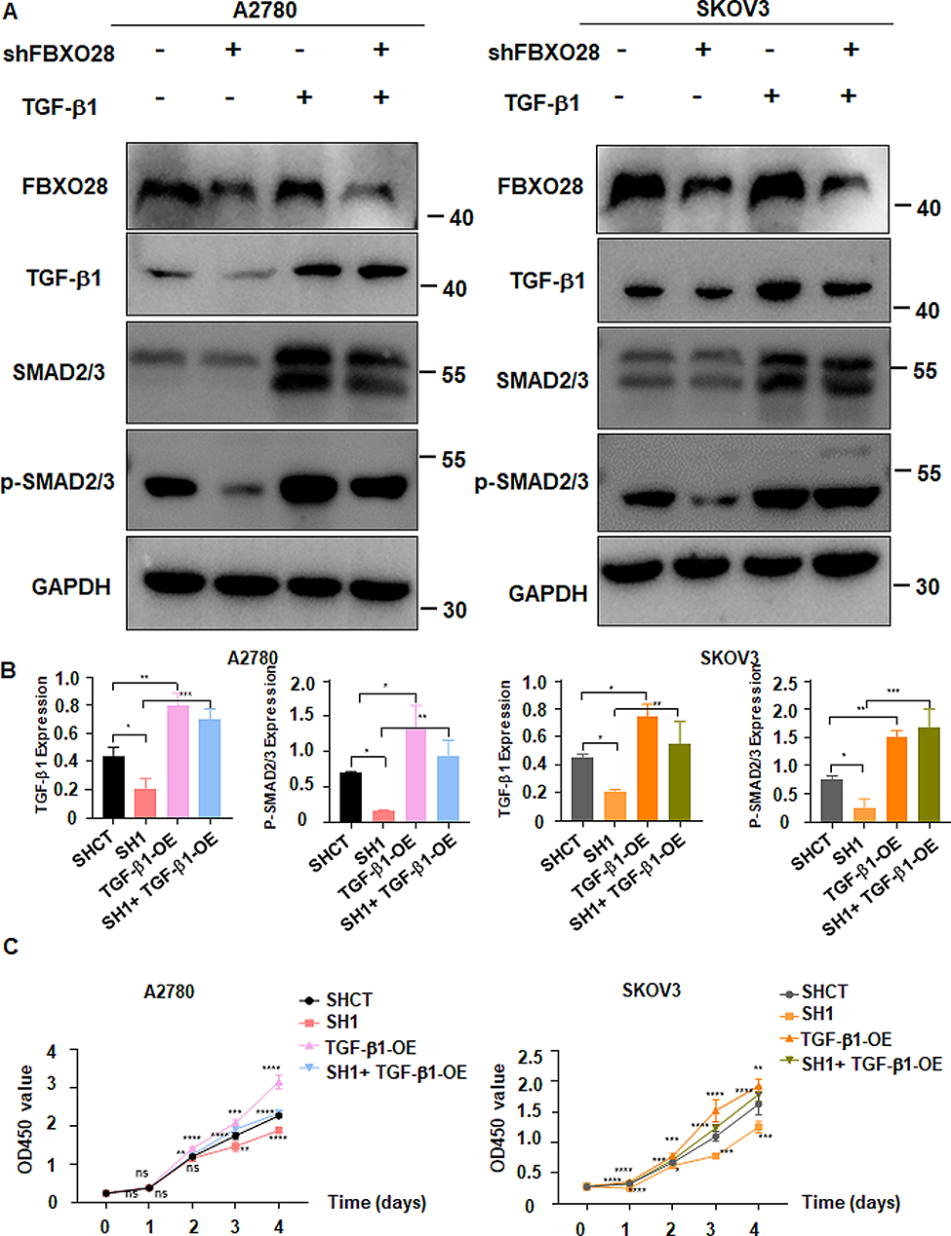
TGF-b1 overexpression rescues FBXO28 knockdown-mediated suppression of cell viability. A. The expression of TGF-b1, Smad2/3 and p-Smad2/3 in ovarian cancer cells was measured by western blotting after FBXO28 knockdown and TGF-b1 overexpression. B. The quantitative analysis results obtained in Figure A. C. A CCK-8 assay showed that overexpression of the TGF-b1 gene reversed the FBXO28 knockdown-mediated decrease in ovarian cancer cell viability. *P < 0.05, **P < 0.01, ***P < 0.001, ****P < 0.0001

**Fig. 7:**
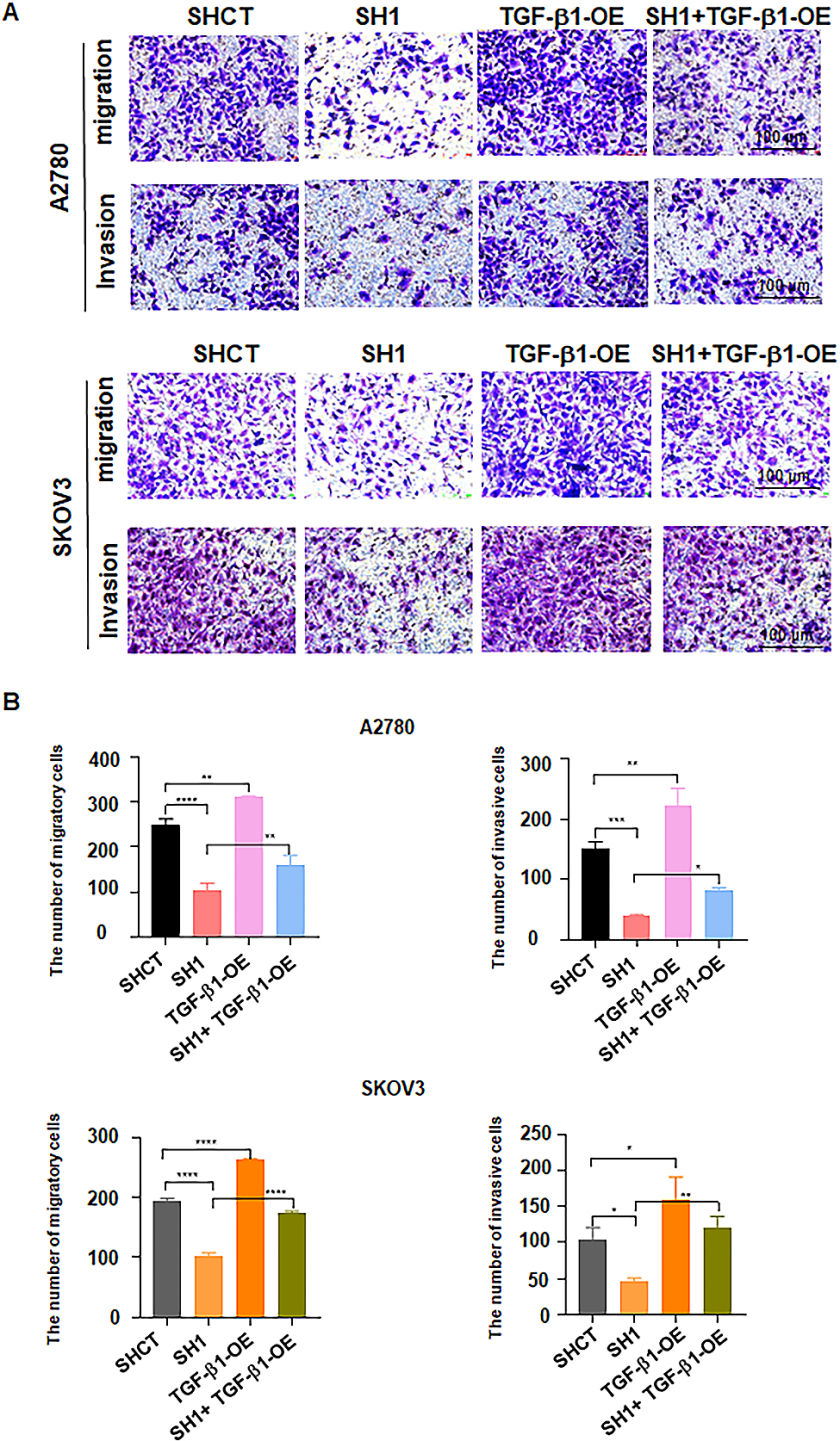
TGF-b1 overexpression rescues FBXO28 knockdown-mediated suppression of migration and invasion. A. Transwell migration and invasion assays were performed in ovarian cancer cells after FBXO28 knockdown and TGF-b1 overexpression. B. The quantitative analysis results obtained in Figure A. *P < 0.05, ** P < 0.01, ***P < 0.001, ****P < 0.0001

To further verify the role of the TGF-b1/Smad2/3 pathway in FBXO28 knockdown-mediated tumor inhibition, we performed rescue experiments in A2780 and SKOV3 cells with FBXO28 knockdown by transfecting a TGF-b1 overexpression plasmid to upregulate TGF-b1 expression. Western blotting revealed that FBXO28 downregulation reduced the activation of p-Smad2/3 in A2780 and SKOV3 cells, which was abrogated by TGF-b1 overexpression (Fig. 6A-B). Moreover, the CCK-8 assay results showed that overexpression of TGF-b1 abolished the FBXO28 knockdown-induced decrease in ovarian cancer cell viability (Fig. 6C, supplementary Table 3). In addition, data from Transwell migration and invasion assays demonstrated that upregulation of TGF-b1 rescued the FBXO28 knockdown-mediated suppression of migratory and invasive abilities in A2780 and SKOV3 cells (Fig. 7A-B). Therefore, the TGF-b1 signaling pathway could be involved in FBXO28-mediated ovarian tumorigenesis.

### FBXO28 knockdown inhibits tumor growth in vivo

**Fig. 8:**
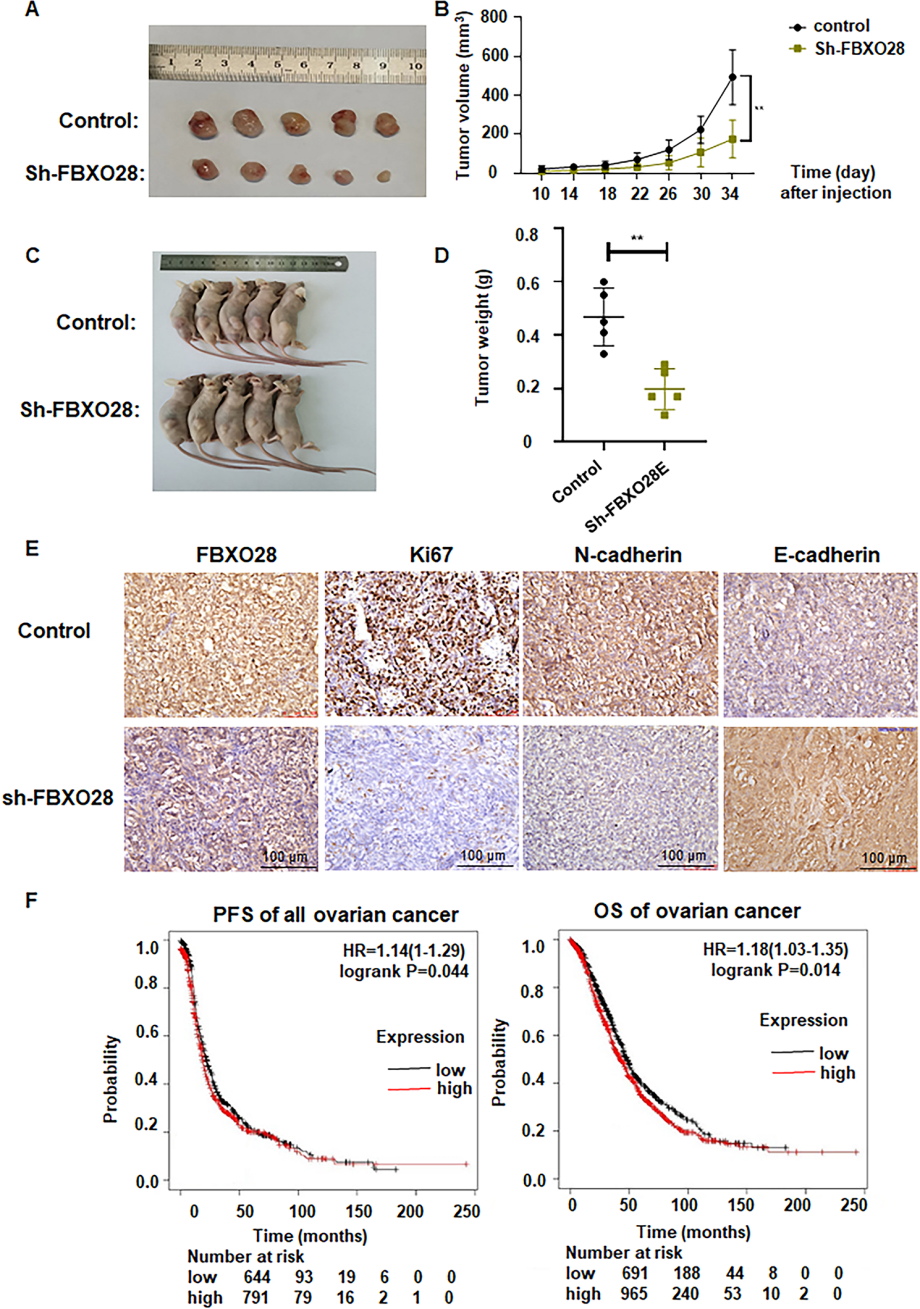
FBXO28 knockdown inhibits tumor growth in vivo. A. Images of xenograft tumors obtained 34 days after injection of SKOV3-knockdown cells or corresponding control cells in nude mice. B. Changes in tumor volume measured at 4-day intervals after injection of SKOV3-knockdown cells or corresponding control cells in nude mice. Tumor volume = (L×W2)/2. CC. Images of the collected graft tumors. D. The weight of each xenograft tumor. E. Representative images of subcutaneous tumor samples from the SKOV3 knockdown and corresponding control groups subjected to immunohistochemical staining for the FBXO28, N-cadherin, E-cadherin and Ki67 proteins. **P < 0.01. F: Left panel: Relationship between FBXO28 expression and PFS in ovarian cancer patients. Right panel: correlation between FBXO28 expression and OS in ovarian cancer patients. **P < 0.01.

To evaluate the cancer-promoting activity of FBXO28 in vivo, we constructed a tumor xenotransplantation model in nude mice by subcutaneously injecting SKOV3 cells with FBXO28 knockdown or corresponding control cells. We found a reduction in tumor weight and volume in the FBXO28-shRNA group compared to those in the control group (Fig. 8A-D). In addition, by performing IHC experiments on tumor tissues, we found that N-cadherin expression was significantly downregulated, and that E-cadherin expression was upregulated in tissues injected with FBXO28-shRNA compared to those injected with control shRNA (Fig. 8E). Consistent with the proliferative behavior of FBXO28-knockdown cells, the expression of Ki67, a proliferative marker, was lower in tissues injected with FBXO28-shRNA than in those injected with the control agent (Fig. 8E). Therefore, the in vivo results further supported that FBXO28 promoted oncogenesis in ovarian cancer.

### FBXO28 expression is linked to poor ovarian cancer prognosis

Lastly, we used the Kaplan‒Meier survival analysis method to evaluate the correlation of between FBXO28 expression and progression-free survival (PFS) and overall survival (OS) in patients with different clinical stages. The results revealed a correlation between the FBXO28 level and ovarian cancer progression (Table 1). The survival curve of FBXO28 levels and ovarian cancer indicated that FBXO28 expression levels could be linked to prognosis of ovarian cancer (Fig. 8F).

**Table 1:** Correlations of FBXO28 expression level with PFS and OS in ovarian cancer patients at different clinical stages. *P < 0.05, **P < 0.01

Stages	PFS			OS		
	Case	HR (95%CI)	*P* value	case	HR (95%CI)	*P* value
1	96	1.57(0.54–4.53)	0.4	74	0.24(0.07–0.76)	0.0089**
2	67	0.62(0.27–1.45)	0.27	61	0.24(0.07–0.91)	0.024*
3	919	1.27(1.09–1.48)	0.0023**	1044	1.31(1.11–1.55)	0.0012**
4	162	1.57(1.05–2.37)	0.028*	176	1.56(1.05–2.31)	0.027*
ALL	1435	1.14(1.0-1.29)	0.044*	1656	1.18(1.03–1.35)	0.014*

## Discussion

The present study demonstrated that FBXO28 expression was associated with poor prognosis in ovarian cancer patients and that the upregulation of FBXO28 was positively correlated with the proliferation, migration and invasion capacity of ovarian cancer cells. Moreover, the expression level of FBXO28 was positively correlated with the expression levels of TGF-b1 and p-Smad2/3. Notably, we observed that the overexpression of TGF-b1 could partially eliminate the inhibitory effects of FBXO28 knockdown on the proliferation, migration and invasion of ovarian cancer cells. Hence, FBXO28 could affect ovarian cancer progression through the TGF-b1 signaling pathway.

In recent years, evidence has implied that FBXO28 may be involved in the occurrence and development of multiple human diseases. For example, FBXO28 variants cause developmental and epileptic encephalopathy, and with severe intellectual disability [31]. It has been found that FBXO28 is needed for the normal mitosis and affects the cell cycle by regulating topoisomerase IIα activity to maintain genome stability [32]. A study showed that sublytic C5b-9 induces glomerular mesangial cell proliferation with the help of FBXO28, which is associated with the development of mesangial proliferative glomerulonephritis [33]. Interestingly, one study reported that FBXO28 mediates its self-ubiquitination of FBXO28, leading to its degradation [34]. By a comprehensive analysis, FBXO28 was found to be highly expressed in pancreatic ductal adenocarcinoma [35]. However, no studies have evaluated FBXO28 in ovarian cancer. Our study not only confirmed that FBXO28 may be associated with the prognosis of ovarian cancer patients, but also analyzed the related molecular mechanisms of FBXO28-mediated ovarian carcinogenesis, helping to elucidate the role of FBXO28 in ovarian cancer.

As a common signaling pathway, TGF-b1, a member of the TGF-b family, is corelated with the occurrence and development of many cancers [36, 37]. The TGF-b1 signaling pathway involves binding of mainly TGF-b1 ligands to their receptors, and the phosphorylated TGF-b1 receptor can activate various signaling pathways, including the Smad2/3, Ras and PI3K pathways [38]. Several studies have shown that TGF-b1 signaling can be associated with the cause and development of cancer by regulating cell migration, the tumor microenvironment and metastatic capacity [39–43]. In ovarian cancer, TGF-b1 has been shown to regulate proliferation, the EMT, the stem cell phenotype, and metastasis [44–46]. Blocking the TGF-b1 pathway inhibits the self-renewal, migration and invasion of stem cells in ovarian cancer [47]. Yeung et al. reported that TGF-b1 governs cell invasion via upregulation of CAF-derived versican in ovarian cancer [24]. In the present study, we found that the upregulation of FBXO28 increased the expression levels of TGF-b1 and p-Smad2/3, increased N-cadherin expression and decreased E-cadherin expression levels. Therefore, FBXO28 exerts its oncogenic function partly by targeting the TGF-b1/p-Smad2/3 axis in ovarian cancer.

However, there are several limitations of our study that should be mentioned and explored in the future. To validate the expression levels of FBXO28 in ovarian cancer tissues, it is necessary to increase the number of ovarian cancer patients. Kaplan‒Meier survival analysis indicated that FBXO28 expression levels could be associated with prognosis of ovarian cancer. Although the statistical significance was observed from the P value, the red line (high FBXO28) and the black line (low FBXO28) crossed each other at more than one-time point, which suggests that there is statistical significance, but no clinical significance. Hence, it is hard to conclude that the expression of FBXO28 was associated with ovarian cancer prognosis, which needs further exploration whether FBXO28 could be a predictor of prognosis of ovarian cancer patients. In addition, how FBXO28, a constituent of the ubiquitin proteasome, regulates the TGF-b1 signaling pathway has not been determined. Moreover, in animal experiments, FBXO28 conditional transgenic or knockout mouse models are ideal tools for determining the role of FBXO28 in ovarian cancer. Taken together, due to the critical oncogenic role of FBXO28 in ovarian cancer, our study indicated that targeting FBXO28 by its inhibitors could be a promising approach for the treatment of ovarian cancer.

### Electronic supplementary material

Below is the link to the electronic supplementary material.

Supplementary Material 1

Supplementary Material 2

Supplementary Material 3

Supplementary Material 4

Supplementary Material 5

## Data Availability

The data in this study are available from the corresponding author on reasonable request.
