# Supplementary material for: FBXO28 promotes cell proliferation, migration and invasion via upregulation of the TGF-beta1/SMAD2/3 signaling pathway in ovarian cancer
Source: BMC Cancer. 2024 Jan 24;24:122. doi: 10.1186/s12885-024-11893-8 (PMC10807113; doi:10.1186/s12885-024-11893-8)
Supplement: Supplementary file 1 — Supplementary Material 1 [file 12885_2024_11893_MOESM1_ESM.pdf]

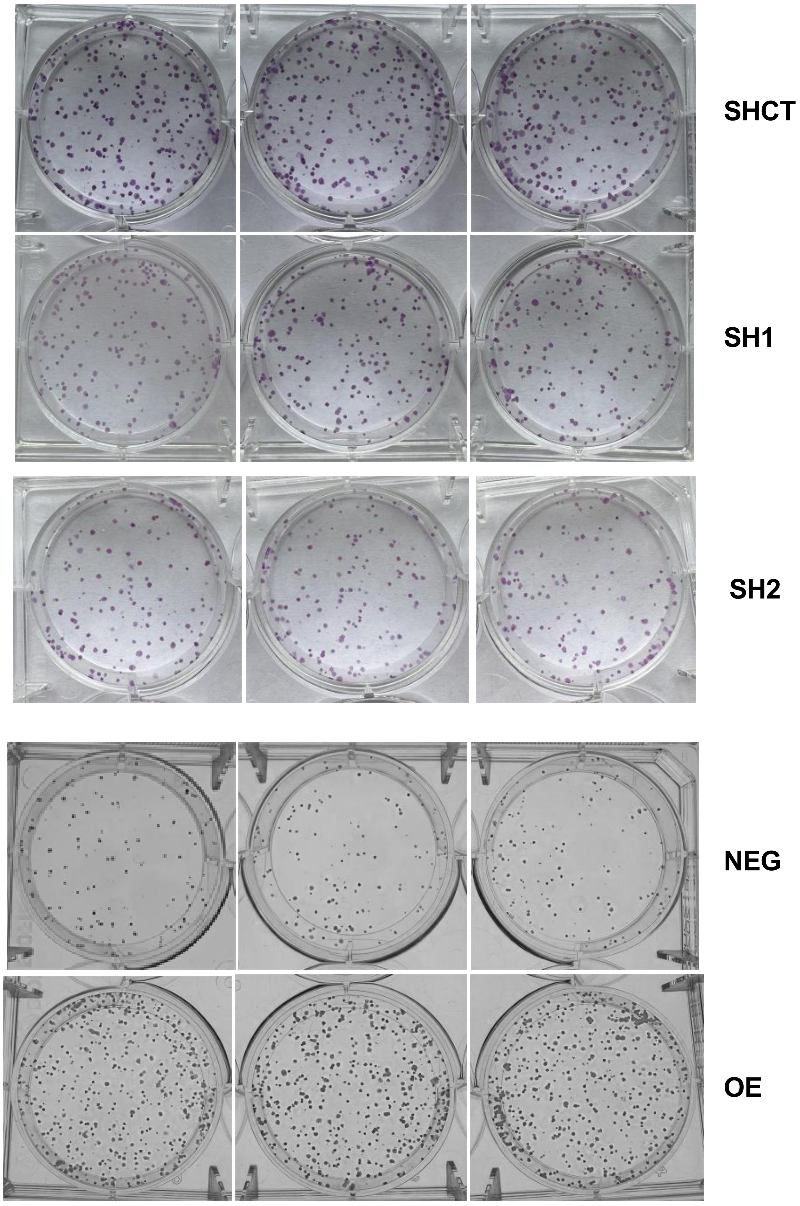

Supplementary figure 1: FBXO28 modulations change colony formation in A2780 cells. FBXO28 knockdown decreased the proliferation capacity of A2780 cells, as determined by a colony formation assay. FBXO28 overexpression increased the proliferation capacity of A2780 cells, as determined by a colony formation assay.
