# Supplementary material for: FBXO28 promotes cell proliferation, migration and invasion via upregulation of the TGF-beta1/SMAD2/3 signaling pathway in ovarian cancer
Source: BMC Cancer. 2024 Jan 24;24:122. doi: 10.1186/s12885-024-11893-8 (PMC10807113; doi:10.1186/s12885-024-11893-8)
Supplement: Supplementary file 2 — Supplementary Material 2 [file 12885_2024_11893_MOESM2_ESM.docx]

|  | Ovarian cancer  （n=10） | Non-ovarian cancer（n=10） |
| --- | --- | --- |
| **Age (years old)** |  |  |
| average | 60 | 56 |
| Range | 52-71 | 46-69 |
| **Histologic type** |  |  |
| Serous | 7 | - |
| Clear cell | 1 | - |
| Endometrioid | 2 | - |
| **Stage** |  |  |
| I | 4 | - |
| II | 1 | - |
| III | 5 | - |
| **Chemotherapy** |  |  |
| Yes | 0 | - |
| No | 10 | - |

Supplementary Table 1: The demographic information of ovarian cancer patients.
