# Supplementary material for: FBXO28 promotes cell proliferation, migration and invasion via upregulation of the TGF-beta1/SMAD2/3 signaling pathway in ovarian cancer
Source: BMC Cancer. 2024 Jan 24;24:122. doi: 10.1186/s12885-024-11893-8 (PMC10807113; doi:10.1186/s12885-024-11893-8)
Supplement: Supplementary file 3 — Supplementary Material 3 [file 12885_2024_11893_MOESM3_ESM.docx]

Supplementary Table 2: The doubling time for Figure 2C-2D.

| Cell types | | Doubling time（hours） |
| --- | --- | --- |
| A2780 cells | SHCT | 34.96 |
|  | SH1 | 45.13 |
|  | SH2 | 47.25 |
| SKOV3 cells | SHCT | 31.06 |
|  | SH1 | 37.92 |
|  | SH2 | 39.20 |
| A2780 cells | NEG | 33.17 |
|  | OE | 29.46 |
| SKOV3 cells | NEG | 36.96 |
|  | OE | 33.96 |
