# Supplementary material for: FBXO28 promotes cell proliferation, migration and invasion via upregulation of the TGF-beta1/SMAD2/3 signaling pathway in ovarian cancer
Source: BMC Cancer. 2024 Jan 24;24:122. doi: 10.1186/s12885-024-11893-8 (PMC10807113; doi:10.1186/s12885-024-11893-8)
Supplement: Supplementary file 4 — Supplementary Material 4 [file 12885_2024_11893_MOESM4_ESM.docx]

Supplementary Table 3: The doubling time for Figure 6C.

| Cell types | | Doubling time (hours) |
| --- | --- | --- |
| A2780 cells | SHCT | 29.16 |
|  | SH1 | 31.72 |
|  | TGF-β1-OE | 25.52 |
|  | SH1+TGF-β1-OE | 28.91 |
| SKOV3 cells | SHCT | 37.35 |
|  | SH1 | 43.94 |
|  | TGF-β1-OE | 34.16 |
|  | SH1+TGF-β1-OE | 36.51 |
