# Supplementary material for: FBXO28 promotes cell proliferation, migration and invasion via upregulation of the TGF-beta1/SMAD2/3 signaling pathway in ovarian cancer
Source: BMC Cancer. 2024 Jan 24;24:122. doi: 10.1186/s12885-024-11893-8 (PMC10807113; doi:10.1186/s12885-024-11893-8)
Supplement: Supplementary file 5 — Supplementary Material 5 [file 12885_2024_11893_MOESM5_ESM.pdf]

Figure 1E

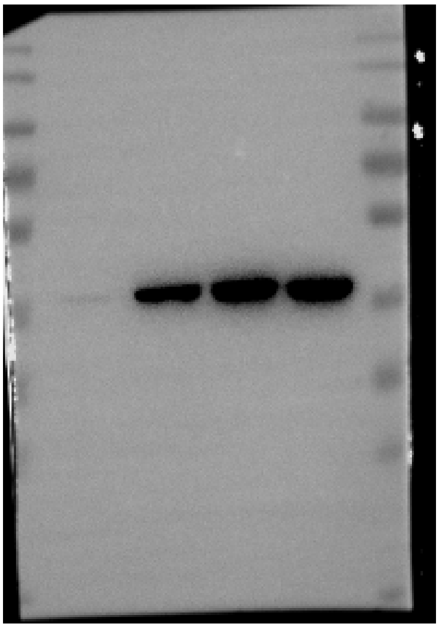

FBXO28

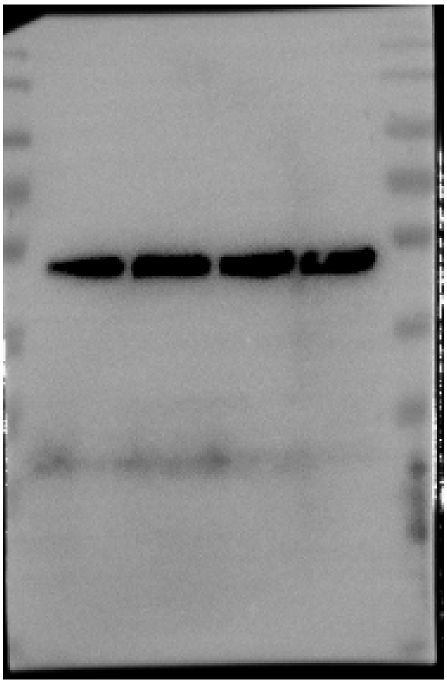

Tubulin

Figure 2A

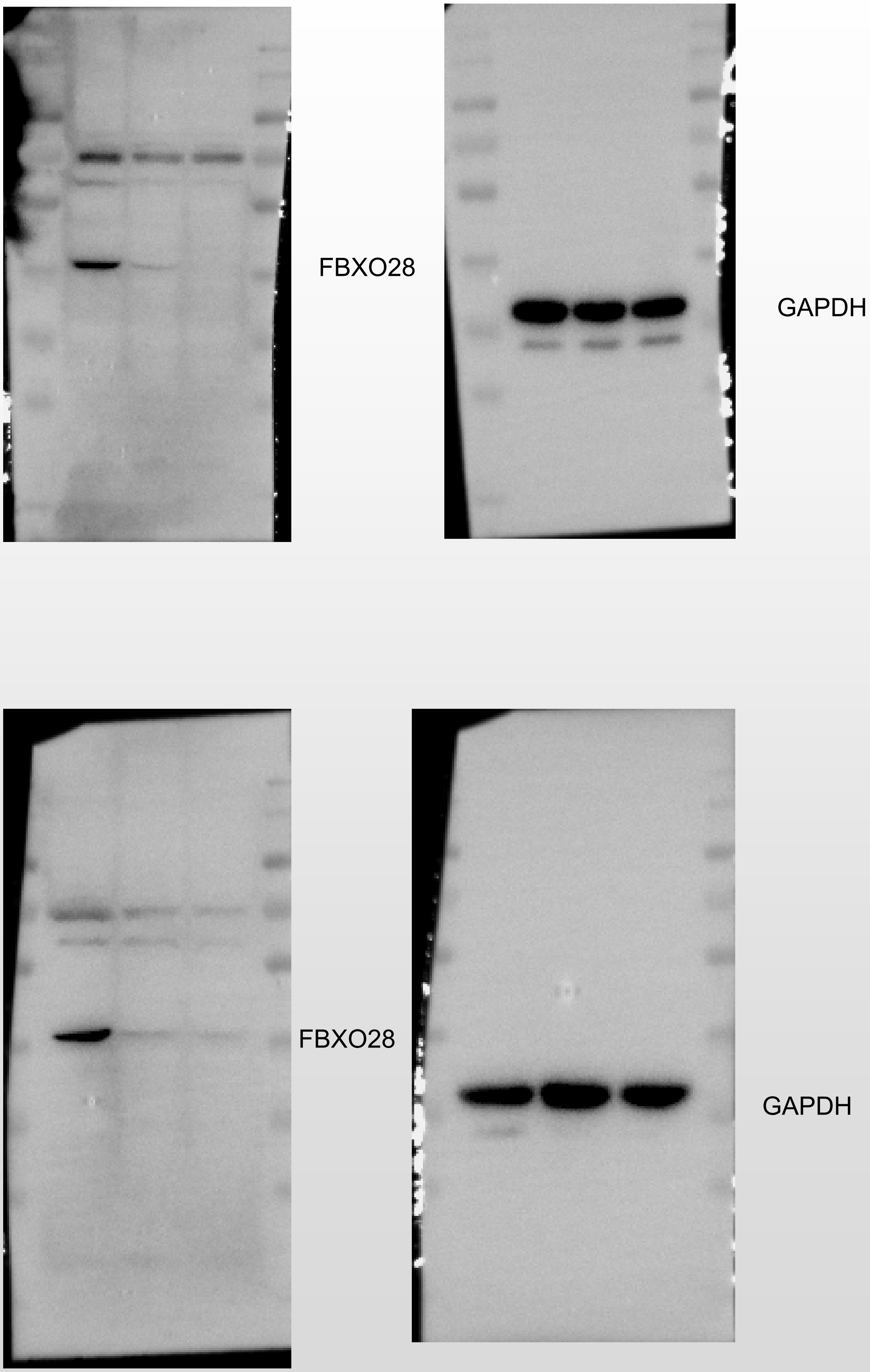

Figure 2B

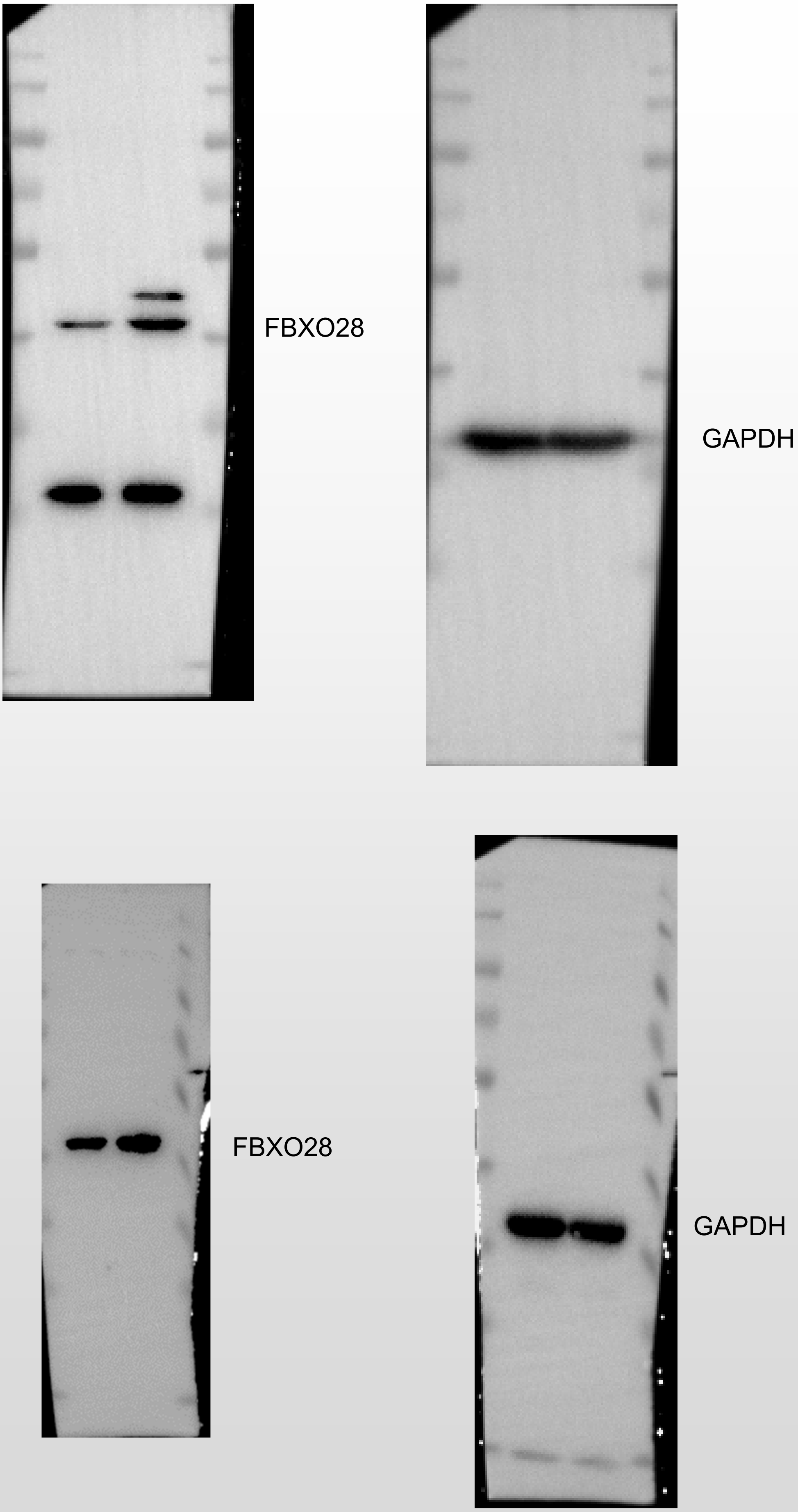

Figure 4B

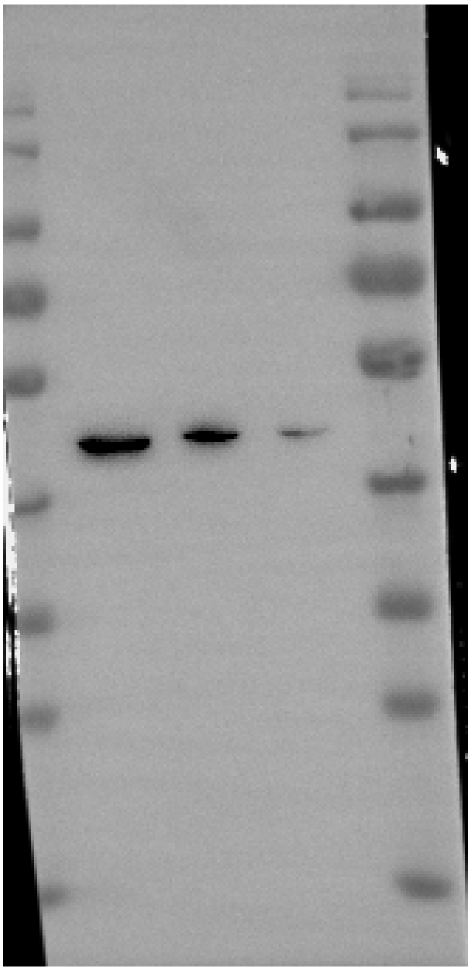

TGF-β1

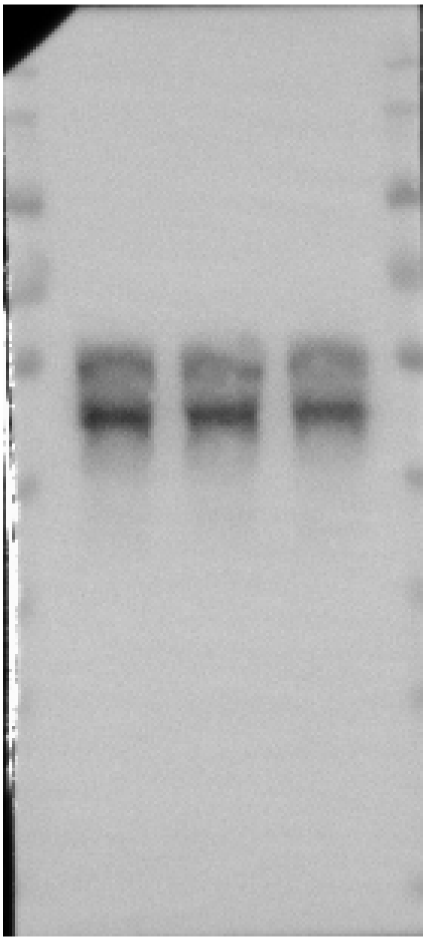

SMAD2/3

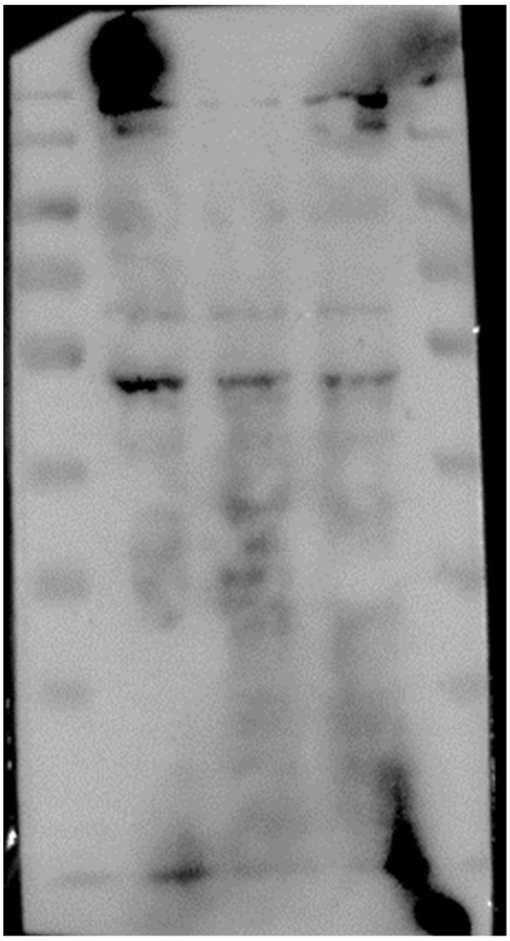

P-SMAD2/3

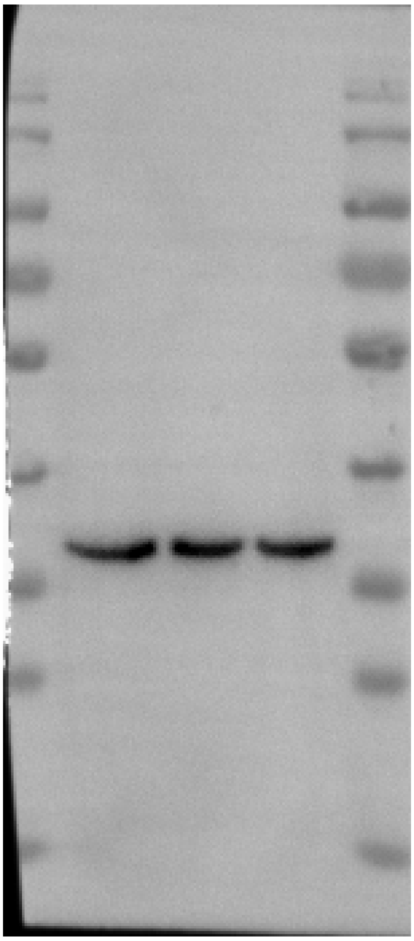

GAPDH

Figure 4C

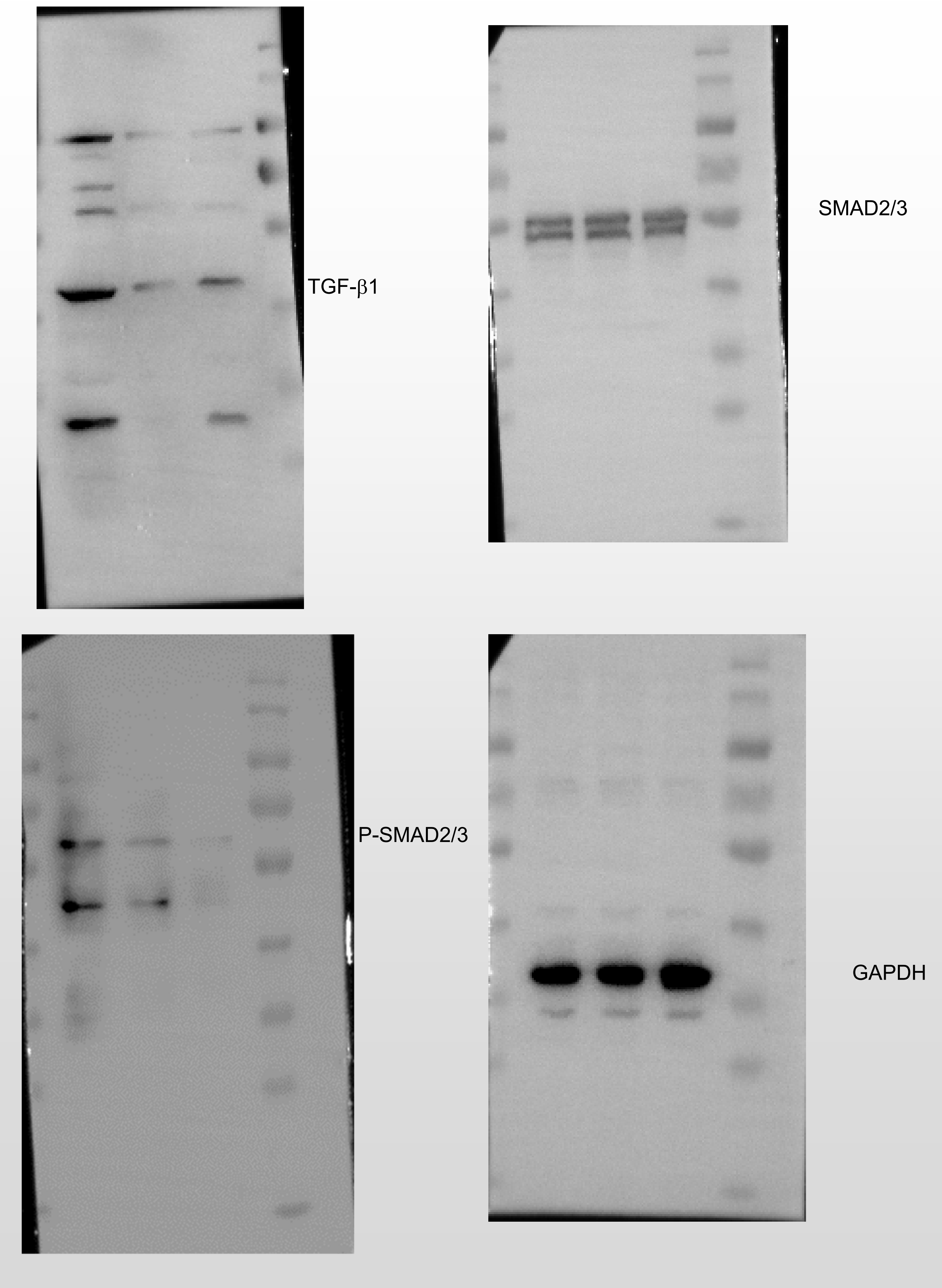

Figure 4E left

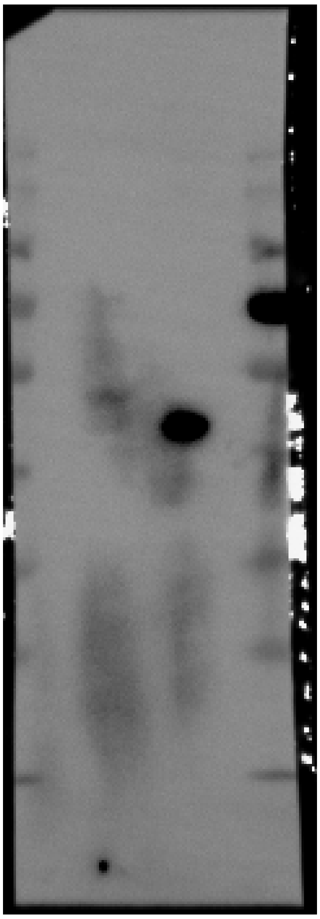

TGF-β1

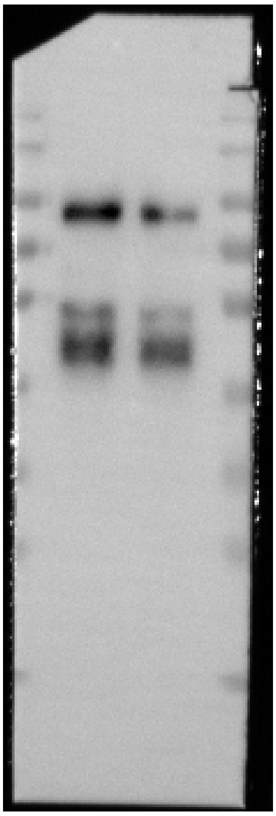

SMAD2/3

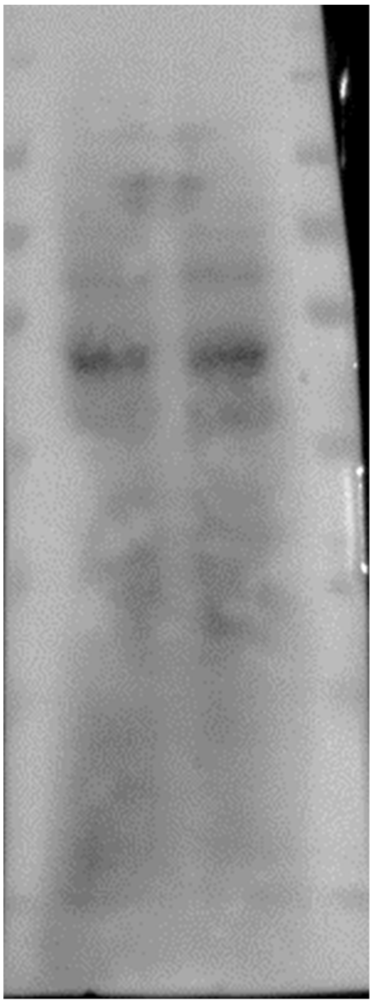

P-SMAD2/3

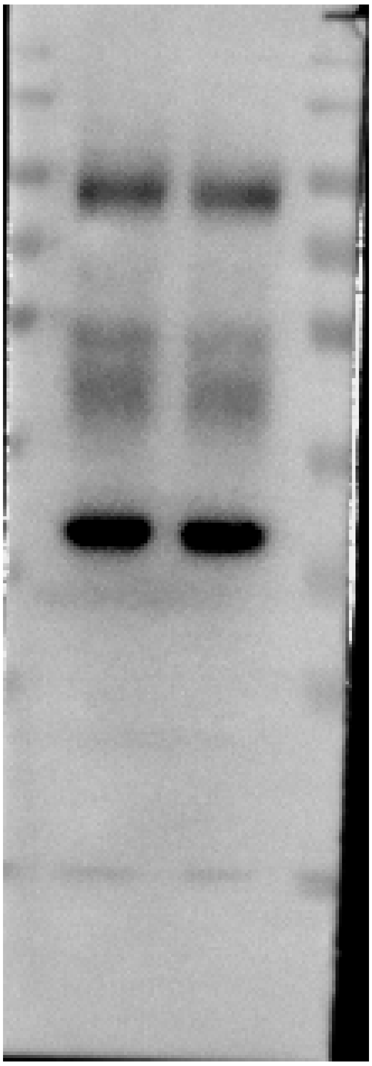

GAPDH

Figure 4E right

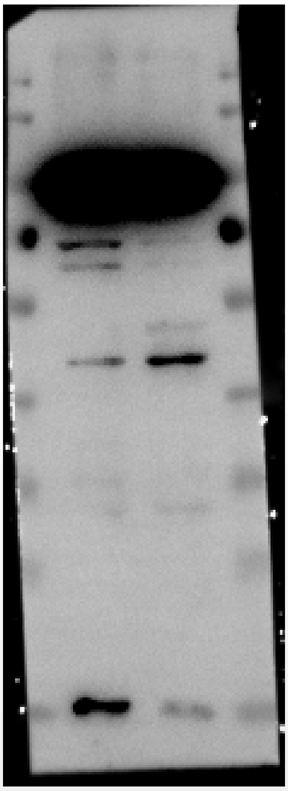

TGF-β1

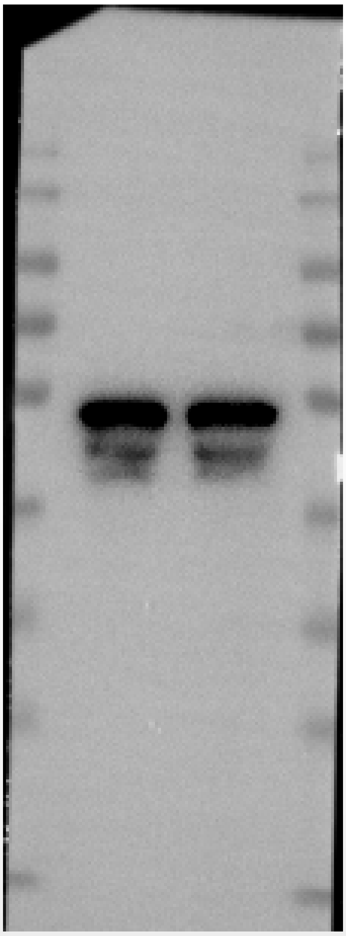

SMAD2/3

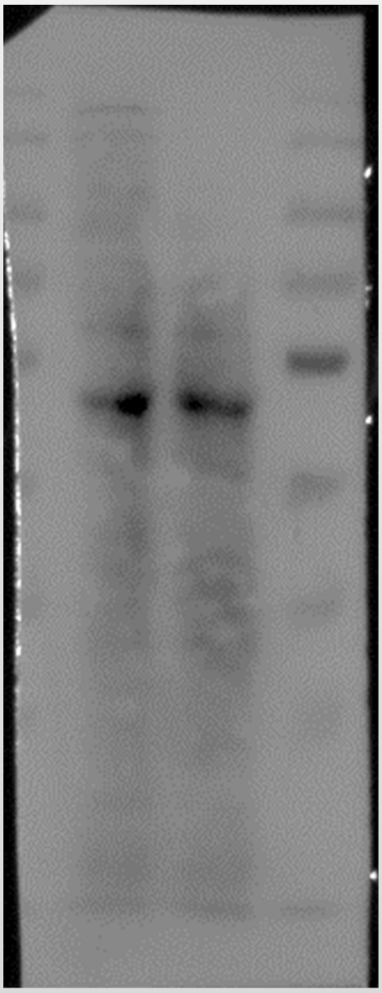

P-SMAD2/3

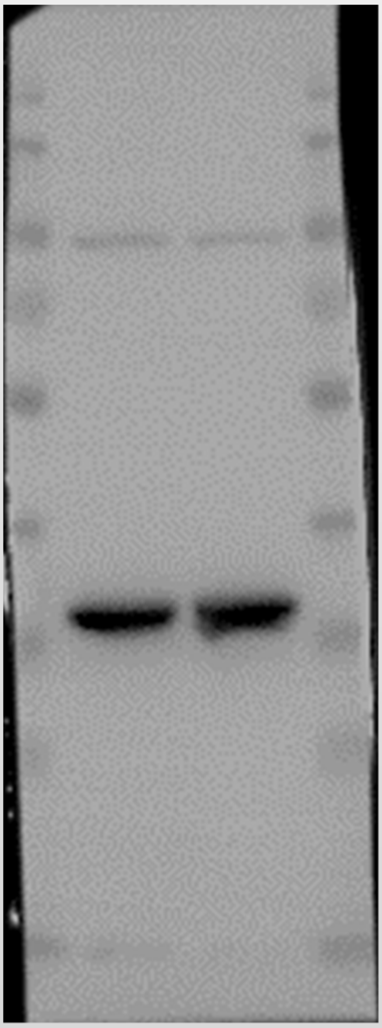

GAPDH

Figure 4B

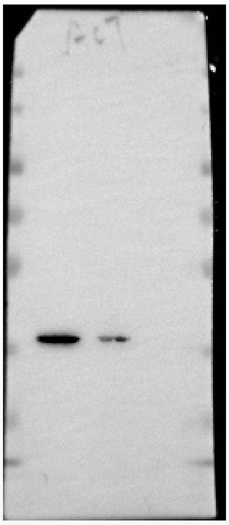

FBXO28

Figure 4C

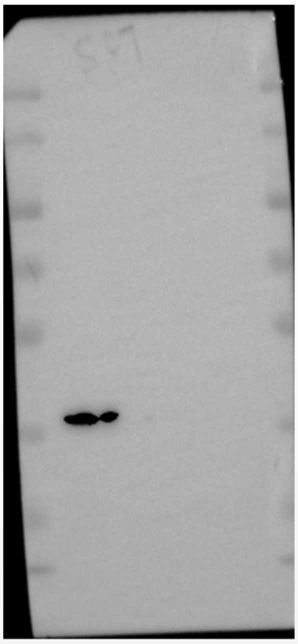

FBXO28

Figure 4E

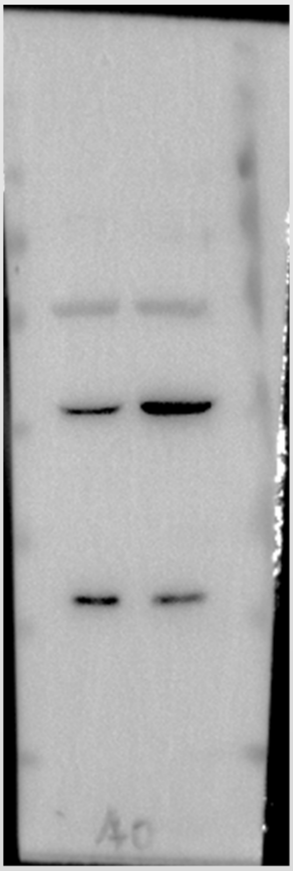

FBXO28

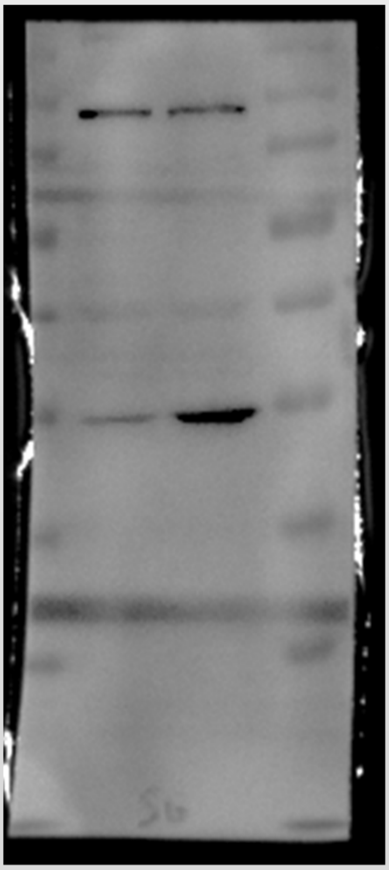

FBXO28

Figure 5A

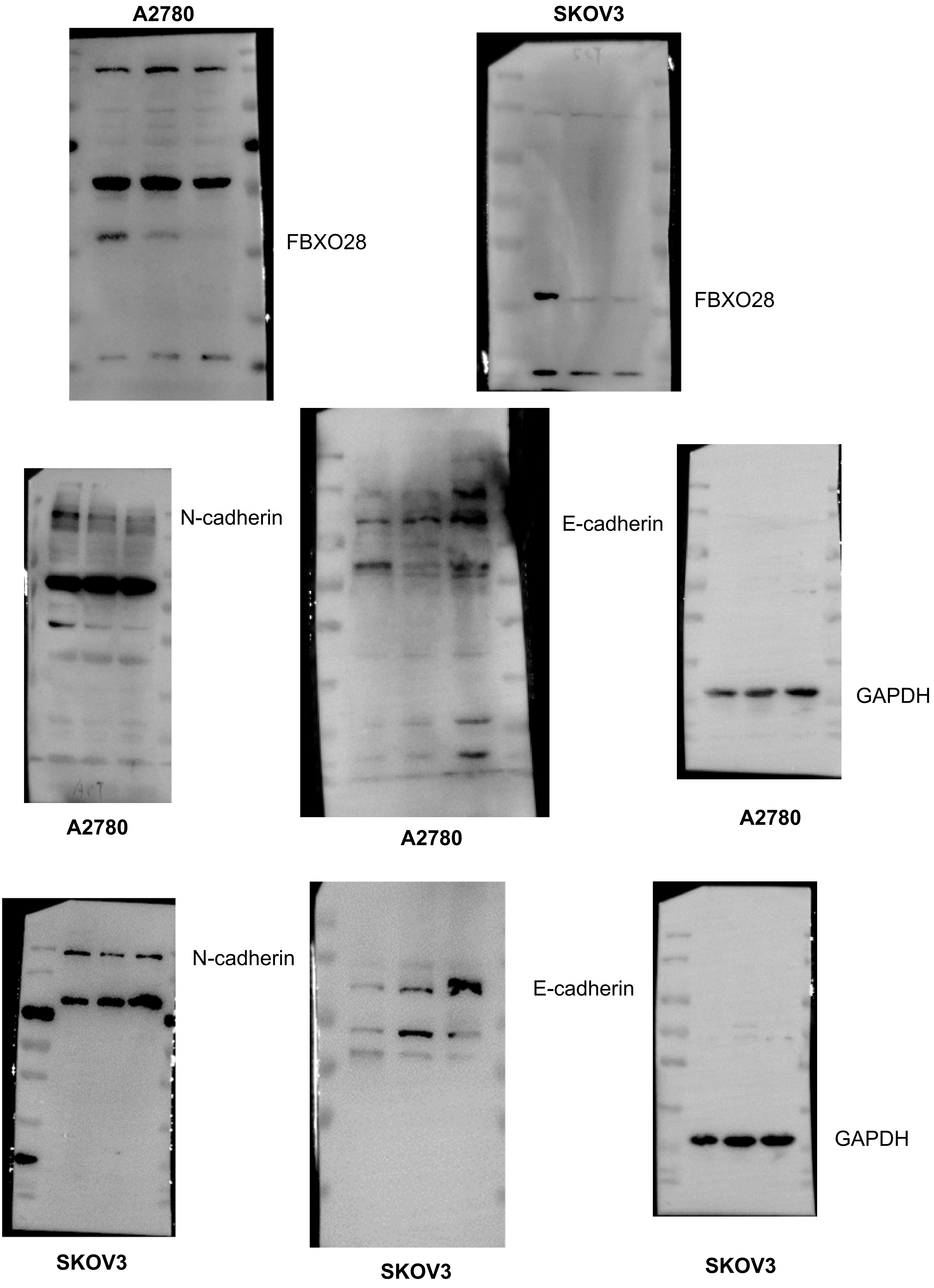

A2780
SKOV3
FBXO28
FBXO28
N-cadherin
E-cadherin
GAPDH
A2780
A2780
A2780
N-cadherin
E-cadherin
GAPDH
SKOV3
SKOV3
SKOV3

Figure 5C

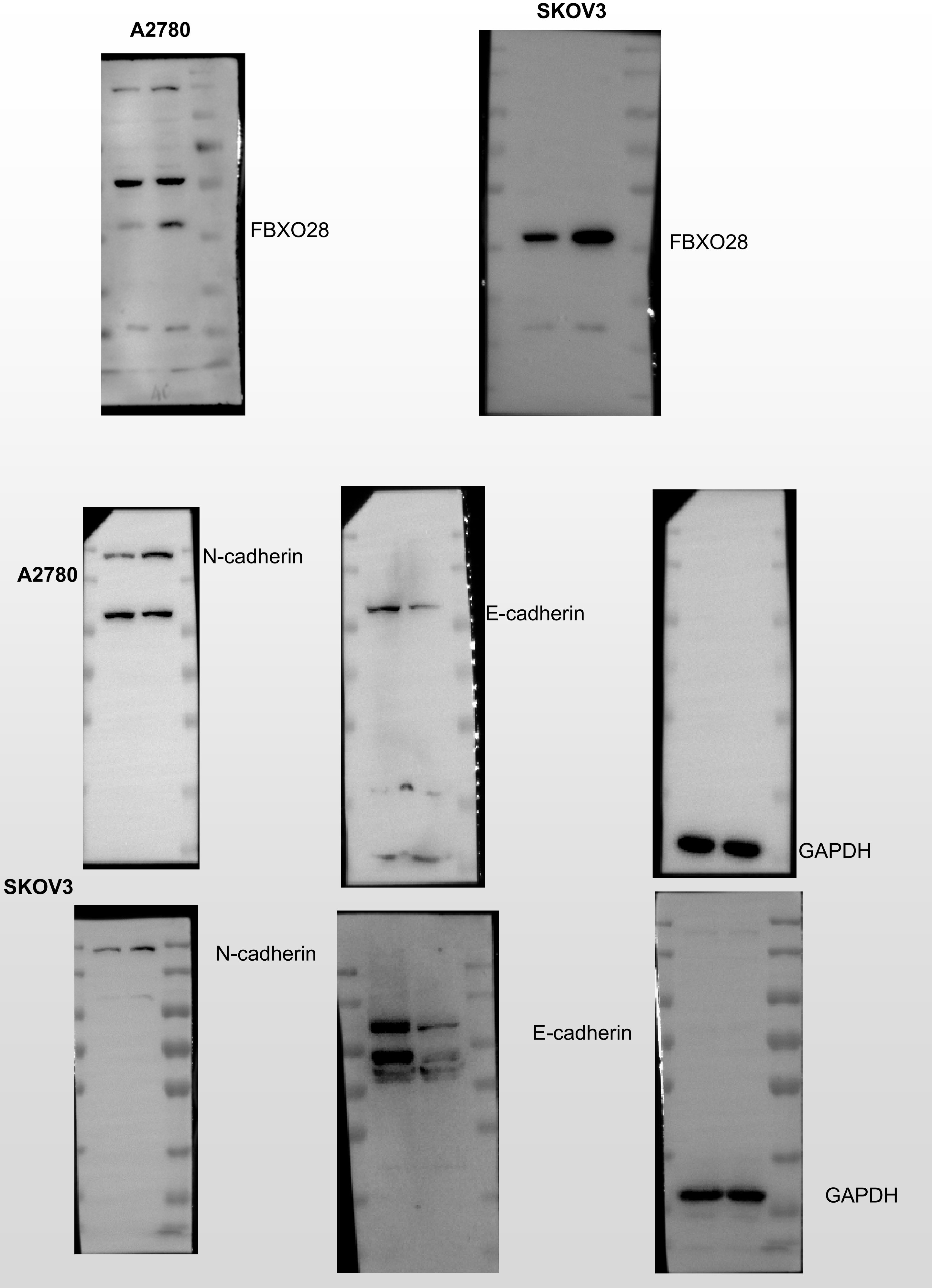
A2780
FBXO28
SKOV3
FBXO28
A2780
N-cadherin
E-cadherin
GAPDH
SKOV3
N-cadherin
E-cadherin
GAPDH

Figure 6A

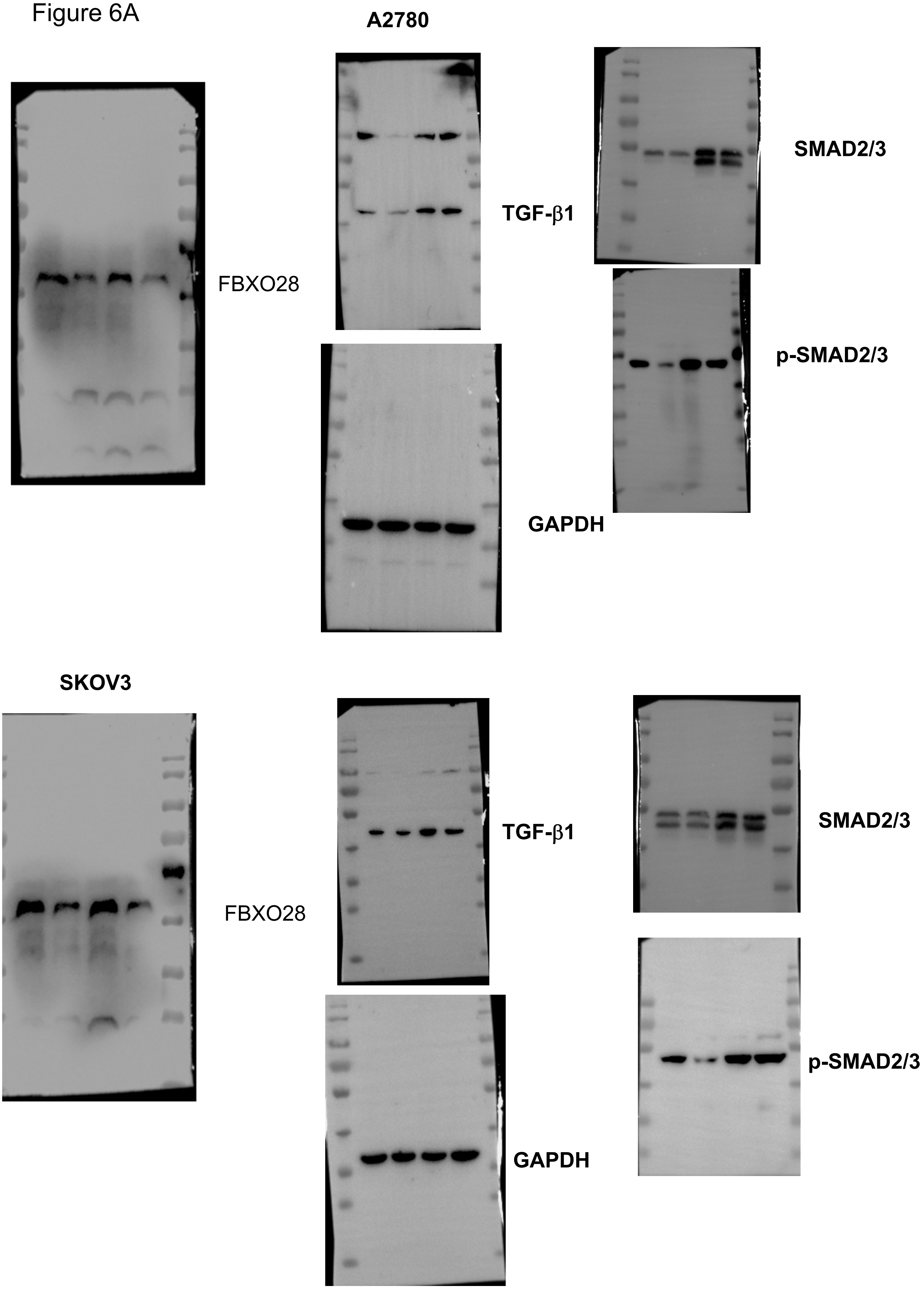

A2780
FBXO28
TGF-β1
GAPDH
SMAD2/3
p-SMAD2/3
SKOV3
FBXO28
TGF-β1
GAPDH
SMAD2/3
p-SMAD2/3
